# Clinical OCT computation-modeled prognosis of coronary plaque progression with validation by fusogenic macrophage nano-targeting

**DOI:** 10.1186/s40580-026-00567-9

**Published:** 2026-07-30

**Authors:** Sewoom Baek, Hyun-Su Ha, Seyong Chung, Ji Youn Lee, Hyeongyun Choi, Chansik Kim, Seok Joon Lee, Seul-Gee Lee, Yong-Joon Lee, Jung-Sun Kim, Hak-Joon Sung

**Affiliations:** 1 Department of Biomedical Engineering, Yonsei University College of Medicine, 50-1 Yonsei-ro, Seodaemun-gu, Seoul, 03722 Republic of Korea; 2 Division of Cardiology, Severance Hospital, Yonsei University College of Medicine, 50-1 Yonsei-ro, Seodaemun-gu, Seoul, 03722 Republic of Korea; 3 Department of Plastic and Reconstructive Surgery, Asan Medical Center, University of Ulsan College of Medicine, 388-1 Pungnap-dong, Sonpa-gu, Seoul, 138-736 Republic of Korea

**Keywords:** Coronary plaque progression, Optical coherence tomography, Computational fluid dynamics, Fusogenic macrophage derived nanovesicle, Plaque-on-a-chip, Ex vivo OCT imaging

## Abstract

Coronary plaque progression often deviates from the diagnostic range of clinical examinations, resulting in the need for emergent follow-ups and increased mortality among outlier patients. This study first analyzes clinical data of fractional flow reserve, optical coherence tomography (OCT), and computed tomography from 180 patients over a 9 years period, with an average follow-up of 2 years. When the plaque progression and healthy control groups were compared in a 1:1 match (n = 10 each), analysis of single artery images failed to detect plaque progression. Therefore, 100–200 image frames were used to reconstruct patient-specific coronary anatomy using computational fluid dynamics, enabling prognostic assessment of plaque progression. The results suggest that steep plaque slopes promote plaque progression by increasing shear stress and hemodynamic disturbance accompanied by greater macrophage recruitment than gentle slopes, which was validated by a microfluidic model. Moreover, increased activation and fusogenic potential of macrophages in steep plagues justified the development of fusogenic macrophage (FM)-vesicles to detect plaque progression. The fusion potential enables FM-vesicles to self-target fusogenic macrophages more efficiently than in vitro compared to macrophages, showing superiority over liposomes and macrophage-vesicles. The membrane fusion mechanism facilitates lysosomal escape, allowing prolonged cytosolic retention without degradation. Rabbit carotid ligation was used to produce steep and gentle plaques by controlling the incision direction. When gold nanoparticles were loaded into FM-vesicles and injected into these arteries ex vivo, OCT accurately imaged the plaque slope and detected changes in signal intensity. This study presents the translational potential of FM-vesicles for clinical application by reducing diagnostic outliers in the detection of plaque progression.

**Graphical abstract:**

**Figure:**
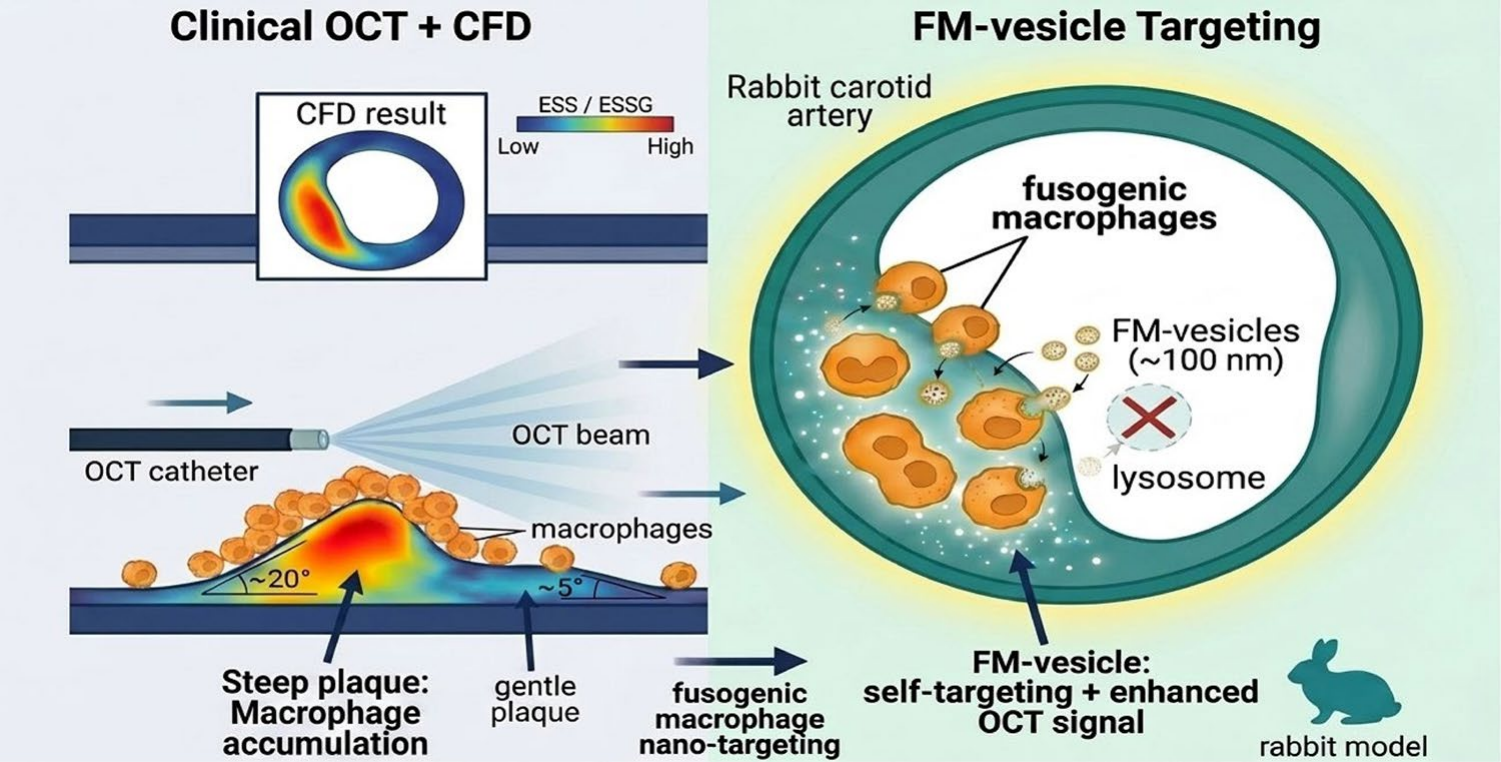

**Supplementary Information:**

The online version contains supplementary material available at 10.1186/s40580-026-00567-9.

## Introduction

Coronary artery disease remains a leading cause of morbidity and mortality worldwide. Notably, acute coronary syndrome often arises from plaques whose anatomical or physiological characteristics are not identified as high-risk by standard examination methods [1, 2], despite continuous advancements in coronary imaging and physiological assessment. Consequently, a subset of patients still experiences rapid plaque progression and rupture, even in the presence of stable angina, preserved fractional flow reserve (FFR), and no apparent stenosis progression [3–5]. This persistent challenge in clinical practice likely stems from the dynamic and heterogeneous nature of atherosclerotic plaque morphology, which can induce abnormalities in local hemodynamics and immune cell behavior [6]. In this context, current clinical imaging modalities require substantial enhancement to more precisely visualize plaque structures and detect pathogenic parameters relevant to vascular remodeling.

When hemodynamic forces are abnormally altered, with elevated of shear rates, endothelial shear stress (ESS), and steep spatial gradients (ESSG), biomechanical cues trigger pathogenic responses such as endothelial cell (EC) dysfunction, monocyte recruitment, and macrophage activation, collectively leading to local inflammation and plaque vulnerability [7]. As a key hemodynamic regulator, the complex geometry of coronary arteries can disturb blood flow, elevate ESS, and disrupt EC junctions [8, 9], thereby promoting lipid deposition. Consequently, the cascade of vascular remodeling progresses from initial vascular inflammation [10] to smooth muscle cell (SMC) migration and subsequent neointimal formation [11]. Persistent ESS abnormalities at stenotic regions further enhance macrophage fusion potential, resulting in the formation of multinucleated giant cells that accelerate plaque progression and instability [12, 13]. Therefore, precise analysis of hemodynamic alterations is essential to detect early plaque progression and improve the initial diagnosis of acute coronary syndrome.

As a leading advancement in intracoronary imaging, optical coherence tomography (OCT) provides high-resolution visualization of plaque structures and their association with vulnerability [14, 15]. However, current OCT modalities remain limited by signal attenuation, resolution constraints, and the inability to directly quantify molecular and cellular activities within plaques without histopathological validation [16]. To overcome these limitations, hybrid and molecular contrast OCT technologies are being developed using nanoparticle-based probes, enabling enhanced compositional and functional imaging [17, 18]. This technological progression highlights the urgent need for nano-agents capable of diagnosing plaque progression under OCT visualization. Furthermore, integrating computational fluid dynamics (CFD) models with OCT and computed tomography (CT) imaging allows for patient-specific reconstruction of coronary anatomy, facilitating detailed analysis of plaque geometry and hemodynamic alterations predictive of vulnerability [19, 20]. As a key indicator of progressive plaque instability, macrophage accumulation within plaques has been visualized in vivo through OCT-based image processing methods [21, 22], suggesting strong potential for clinical translation.

Macrophages within atherosclerotic plaques exhibit extensive functional heterogeneity and play central roles in both disease progression and resolution. As a regulatory phenotype, M1 macrophages are classically activated by pro-inflammatory signals to secrete destructive cytokines and matrix metalloproteinases (MMPs), thereby destabilizing plaques and increasing the risk of rupture and thrombosis [23, 24]. In contrast, alternatively activated M2 macrophages promote anti-inflammatory responses, tissue repair, and plaque stabilization. Notably, interleukin-4 (IL-4) enhances the fusogenic activity of macrophages, promoting cell–cell fusion to form multinucleated giant cells [25], which represent an advanced inflammatory phenotype. This fusogenic potential highlights a possible diagnostic marker of plaque progression and provides the conceptual foundation of this study, leveraging fusogenic macrophages to generate nano-vesicles capable of self-targeting and loading of OCT imaging agents. In this context, cell membrane-derived nanovesicles represent a relevant engineering strategy, as they retain the biological identity and homing capacity of their parent cells. Extracellular vesicles produced from macrophages and nanoparticles coated with macrophage membrane have been actively explored for targeted drug delivery and diagnostic imaging because of their preferential accumulation in inflammatory lesions and atherosclerotic plaques [26, 27]. In this study, a unique strategy for nanovesicle design was developed by considering the fusion state of activated macrophages rather than merely relying on the cell type. Macrophages are activated from circulating monocytes by adhesion to lesion sites and undergo activation to fuse with one another, producing multinucleated giant cells to elevate phagocytic activities. FM-vesicles were designed to utilize the fusion potential for self-targeting fusogenic macrophages, indicating a technical advance in the field of nanotechnology and extracellular vesicles. FM-vesicles were generated by inducing fusogenic macrophages through IL-4 treatment, and their fusogenic characteristics were validated by the expression of membrane effectors including DC-STAMP, CD44, and E-cadherin. These effectors direct membrane fusion with fusogenic macrophages within plaques, thereby enabling self-targeting. This strategy suggests the feasibility of generating patient-specific FM-vesicles from autologous monocytes, offering a practical advantage for diagnostic application by avoiding the risk of immune rejection [28]. Notably, as macrophage fusion is fundamental driver of plaque progress, targeting this fusion process refines the current diagnostic paradigm by informing the progressive risk of pathological vascular remodeling.

Accordingly, this study aims to develop a clinically translatable tool for diagnosing plaque progression. First, coronary examination data using FFR, OCT, and CT angiography were analyzed from 180 patients presenting with chest pain, with an average follow-up period of two years. To address the limitation of single-frame OCT analysis in detecting plaque progression, 100–200 image frames were applied to CFD modeling to reconstruct patient-specific 3D coronary geometries. Comparative analysis between plaque progression and matched healthy control groups (n = 10 each) revealed that steep plaque slopes serve as key diagnostic markers by inducing abnormal hemodynamics. Specifically, CFD simulations demonstrated that steep slopes significantly elevate ESS and ESSG, leading to macrophage accumulation and inflammatory activation within plaques. These computational findings were validated using a microfluidic plaque-on-a-chip model. Finally, the fusogenic potential of macrophages was utilized to engineer self-targeting nano-vesicles, whose ability to enhance OCT imaging of steep plaques was verified in a rabbit carotid ligation model, underscoring the translational potential of this approach.

## Results

### Analysis of plaque slopes in patient coronary OCT through CFD modeling

**Fig. 1:**
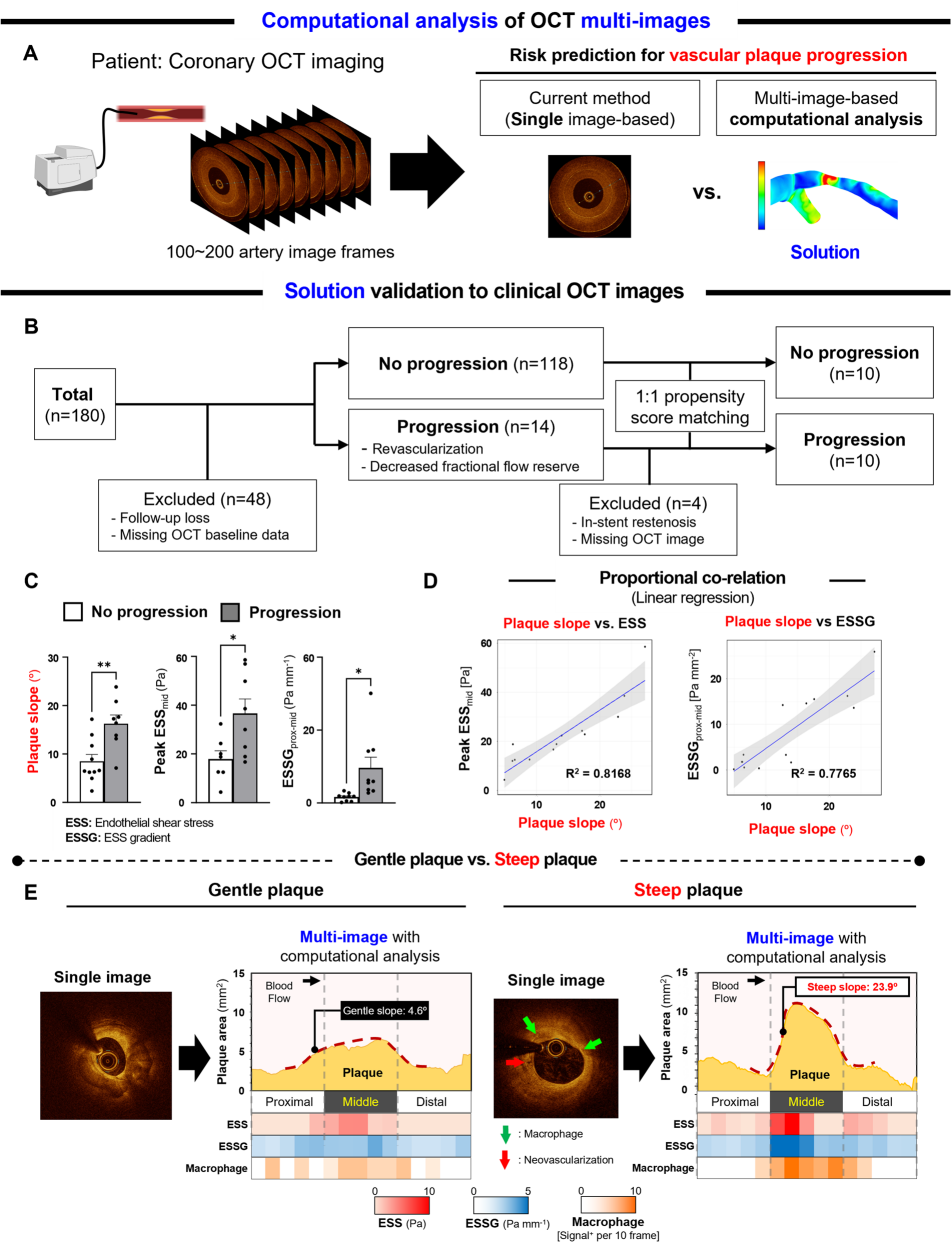
Prognosis of stenotic progression by analyzing plaque slopes of patient coronary arteries through CFD modeling using hundreds of OCT frames. **A** Hundreds of OCT image frames from patient coronary arteries were reconstructed into a multi-image platform to predict patient-specific plaque progression. This approach overcomes the limitations of single-image analysis, enabling identification of plaques with vulnerable potential. **B** In a clinical cohort of 180 patients, 48 were excluded due to loss to follow-up, missing OCT data, or in-stent restenosis. Of the remaining patients, 10 exhibited vessel formation with decreased FFR, indicating plaque progression. A 1:1 propensity score matching was applied to compare the progression and no-progression groups (n = 10 each). **C** Computational analysis using hundreds of OCT images shows that the progression group exhibits significantly higher plaque slope, peak ESS_mid_, and ESSG_prox-mid_ density compared to the no progression group. **D** Linear regression analysis demonstrates that the incremental plaque slope is strongly correlated with peak ESS_mid_ (R^2^ = 0.8168) and ESSG_prox-mid_ (R^2^ = 0.7765). **E** Comparison of FFR records over a 2-year follow up shows that a gentle 4.6° plaque (Patient ID: F231) maintains stable patency, whereas a steep 23.9° plaque (Patient ID: F252) displays elevated macrophage infiltration and neovascularization, with increased ESS and ESSG in the middle plaque region where macrophages are densely localized. Data are presented as mean ± standard deviation (n: dots on each group, independent biological replicates). Statistical significance was determined using a two-sided unpaired t-test for comparisons between two test groups (*p < 0.05, and **p < 0.01)

Conventional analyses based on single OCT images from patient coronary arteries often fail to capture plaque progression, leading to emergency follow-ups and increased mortality risk. To address this issue, hundreds of OCT image frames were reconstructed into a multi-frame analytical platform capable of predicting patient-specific plaque progression (Fig. 1A). This approach allows for the identification of plaques with vulnerable potential, overcoming the limitations of conventional single-image analyses that may misclassify mild lesions. In a clinical cohort of 180 patients (Fig. 1B), 48 patients were excluded due to loss to follow-up, missing OCT data, or in-stent restenosis. Among the remaining participants, 118 patients exhibited no plaque progression. Meanwhile, 10 patients demonstrated revascularization with corresponding decreases in FFR, indicating plaque progression. To ensure comparability between groups, 1:1 propensity score matching was applied to yield two matched cohorts (no progression vs. progression, n = 10 each in Table 1).

**Table 1:** Patient comparison: no versus plaque progression

Characteristics	No progression (n = 10)	Plaque progression (n = 10)	*P* value
Baseline characteristics			
Age, years	61.0 ± 9.9	64.0 ± 5.8	0.424
Gender, Male	9 (90.0%)	8 (80.0%)	1.000
BMI	26.5 ± 3.5	24.4 ± 1.8	0.128
Hypertension	7 (70.0%)	8 (80.0%)	1.000
Diabetes	4 (40.0%)	6 (60.0%)	0.655
Hypercholesterolemia	4 (40.0%)	6 (60.0%)	0.655
Current smoking Angiography parameters	4 (40.0%)	1 (10.0%)	0.303
Pre-FFR	0.91 ± 0.06	0.89 ± 0.04	0.326
Inlet contrast velocity (m s^−1^)	15.7 ± 5.6	16.6 ± 7.2	1.000
Vessel			0.289
LAD	1 (10.0%)	4 (40.0%)	
LCX	4 (40.0%)	4 (40.0%)	
RCA	5 (50.0%)	2 (20.0%)	
OCT parameters			
DS%	51.5 ± 18.4	55.9 ± 13.4	0.554
Lesion length	14.3 ± 3.8	14.6 ± 5.0	0.684
MLD	2.92 ± 2.14	2.41 ± 0.910	0.450
Proximal LA	2.4 [1.1–3.9]	2.0 [1.6–3.1]	0.728
Distal LA	8.1 [6.1–10.3]	8.0 [5.0–9.0]	0.894
Plaque area	13.0 ± 4.6	13.0 ± 5.8	0.979
Fibrocalcific	5 (50.0%)	4 (40.0%)	1.000
Fibrotic	3 (30.0%)	3 (30.0%)	1.000
Lipid-rich	3 (30.0%)	3 (30.0%)	1.000
Necrotic core	5 (50.0%)	5 (50.0%)	1.000
Microvessels	2 (20.0%)	5 (50.0%)	0.350

*BMI*, body mass index, *FFR*, fractional flow reserve, *LAD*, left anterior descending, *LCX*, left circumflex artery, *RCA*, right coronary artery, *OCT*, optical coherent tomography, *DS*, 3-dimensional angiographic percentage diameter stenosis, *MLD*, minimal lumen diameter, *LA*, luminal area

When patient coronary arteries are imaged by angiography (Additional file 1: Fig. S1A), a gentle plaque slope observed during the first visit is associated with preserved vessel patency after 2 years, in contrast to the total occlusion seen in cases with steeper slopes. The plaque angle is quantified as the arctangent of the area difference between the plaque base and peak, divided by the frame interval in OCT imaging (Additional file 1: Fig. S1B). Cross-sectional OCT images of patient coronary arteries reveal macrophage-rich regions (Additional file 1: Fig. S1C). The number of signal-positive frames within 10 consecutive OCT frames of a plaque is used to quantify macrophage accumulation. There are no significant differences between the two groups in baseline clinical characteristics (age, gender, BMI, hypertension, diabetes, hypercholesterolemia, smoking), angiographic parameters (FFR, inlet contrast velocity, vessel diameter), or OCT-derived features (percent diameter stenosis, lesion length, minimal lumen diameter, luminal area, plaque area, and composition including fibrocalcific, fibrotic, lipid-rich, necrotic core, and microvessel regions) (Table 1).

However, computational analysis using hundreds of OCT image frames (Fig. 1C) reveals that the progression group exhibits significantly greater increases in plaque slope, peak ESS_mid_, and ESSG_prox-mid_ density compared to the no-progression group. In the linear regression analysis (Fig. 1D), the incremental plaque slope shows strong positive correlations with both peak ESS_mid_ (R^2^ = 0.8168) and ESSG_prox-mid_ (R^2^ = 0.7765). Macrophage accumulation is also significantly higher in progression lesions, as indicated by an increased number of macrophage-positive frames (5.8) in the middle plaque region compared to the no-progression group (2.0 in Table 2). Furthermore, comparison of FFR records between the two groups over a 2-year follow-up (Fig. 1E) demonstrates that a gentle 4.6° plaque (Patient ID: F231) maintains a stable patency, while a steep 23.9° plaque (Patient ID: F252) shows high-risk features, including macrophage infiltration and neovascularization, accompanied by elevated ESS and ESSG in the middle plaque region where macrophages are densely localized.

**Table 2:** Hemodynamic parameter

Characteristics	No progression (n = 10)	Plaque progression (n = 10)	*P* value
Baseline characteristics			
Plaque slope	8.47 ± 4.5	18.1 ± 9.6	0.002
Peak ESSprox [Pa]	7.6 [4.7–15.6]	4.9 [3.2–10.0]	0.719
Peak ESSmid [Pa]	18.8 [12.6–22.2]	34.2 [21.9–57.3]	0.040
Peak ESSdist [Pa]	14.3 ± 9.8	21.7 ± 15.9	0.182
Mean ESSGprox-mid [Pa mm^−2^]	1.8 [0.8–2.5]	5.9 [3.6–14.2]	0.001
Mean ESSGmid-dist [Pa mm^−2^]	2.0 [0.7–3.2]	8.6 [5.4–16.1]	0.010
Mean number of Macrophage ( +) frame (per 10 frames OCT)			
Proximal macrophage	0.8 [0.2–1.6]	1.2 [0.7–1.8]	0.510
Middle macrophage	2.0 [1.1–3.1]	5.8 [5.2–6.6]	< 0.001
Distal macrophage	0.1 [0–3.1]	0.8 [0.4–1.6]	0.343

In addition, 3D CFD modeling was performed to reconstruct patient-specific coronary geometries and calculate local ESS distributions within the plaque entry region (Additional file 1: Fig. S2). The resulting ESS distributions were projected onto the vessel surface and transformed into 2D heat maps for comparative visualization between patient groups. Non-progressive plaques exhibited relatively uniform ESS distributions with low-intensity heat maps, whereas progressive plaques showed marked ESS fluctuations with elevated heat-map intensities, indicating localized flow disturbances.

### Microfluidic plaque-on-a-chip to validate the slope effect on macrophage activation

**Fig. 2:**
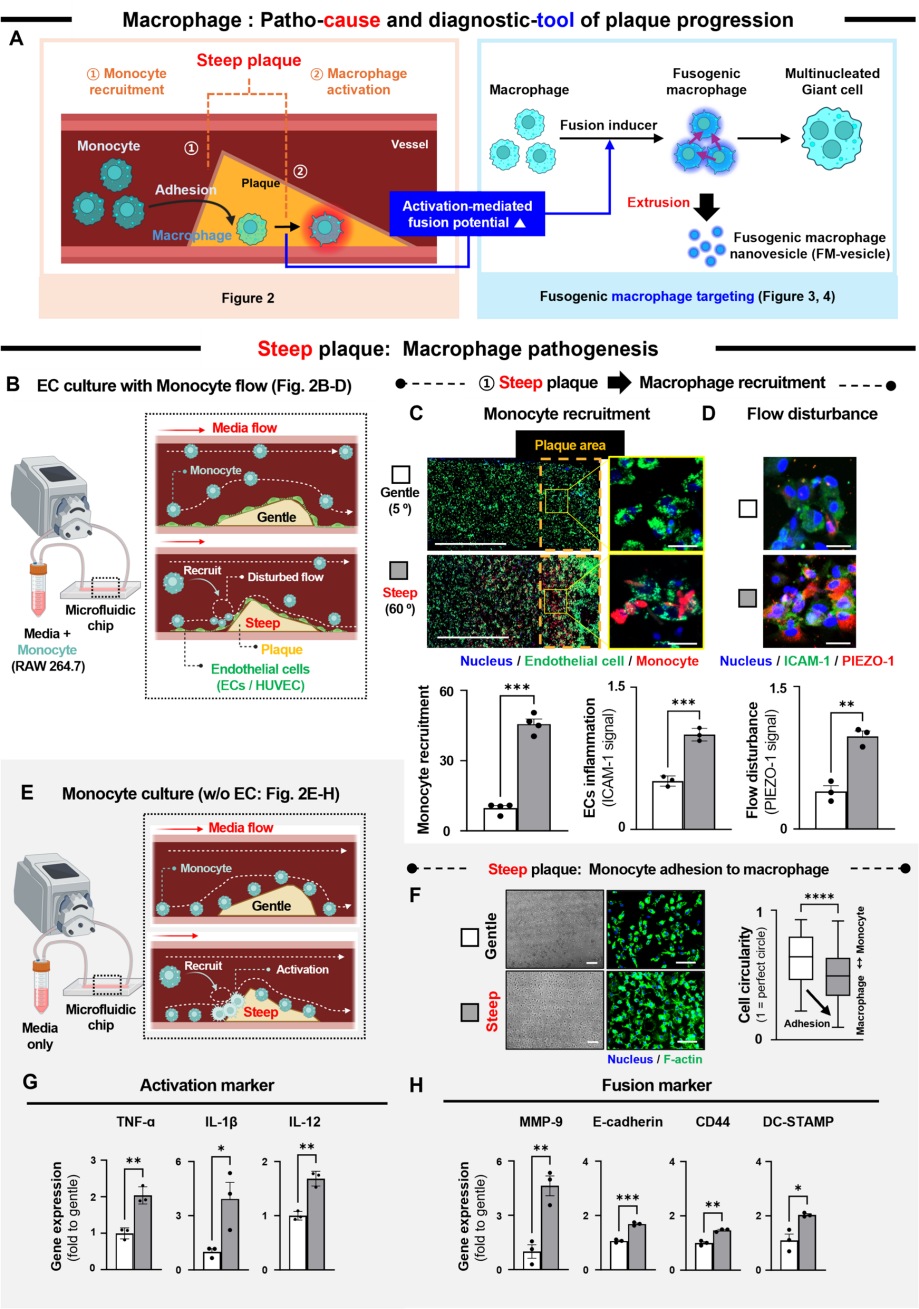
Plaque-on-a-chip to validate the slope effect on recruitment, activation, and fusion of macrophages in microfluidics. **A** Steep plaque slopes disturb hemodynamics, promoting recruitment of monocytes and their activation into macrophages (left). During macrophage activation, fusogenic potential increases inherently or via an inducer (right), enabling the production of fusogenic macrophage (FM)-(nano)vesicles as a diagnostic tool to target vulnerable plaques. **B** The plaque-on-a-chip consists of a microfluidic system in which an endothelial cell (EC, green) monolayer was stabilized after 6 h of culture, followed by perfusion of monocytes (cyan) for 1 day. **C** ECs were stained with DAPI (blue) and DiO (green) to distinguish them from monocytes stained with Dil (red). Co-culture with steep (60°) plaque slopes recruits significantly more monocytes onto ECs compared to gentle (5°) slopes (Scale bars = 1 mm/left and 100 μm/right). **D** Molecular validation shows that ECs increase inflammatory ICAM-1 expression to recruit monocytes, which undergo activation with increased PIEZO-1 expression under hemodynamic disturbance. (Scale bars = 100 μm). **E** An EC-free chip is used to examine direct monocyte responses to flow disturbance under increasing plaque slopes. **F** Monocytes adhere and spread into spindle-like shapes on steep plaques, indicated by decreased circularity compared to those on gentle plaques after 1-day of culture (Scale bars = 100 μm). **G–H** Steep plaques also induce higher expression of macrophage activation markers (TNF-α, IL-1β, IL-12) and fusion-related markers (MMP-9, E-cadherin, CD44, DC-STAMP) compared to gentle plaques. Data are shown as mean ± standard deviation (n: dots represent independent biological replicates in each group). Statistical significance was determined using a two-sided unpaired t-test for comparisons between the two groups (*p < 0.05, **p < 0.01 and ***p < 0.001)

As the steep slope of a plaque disrupts local hemodynamics, monocyte recruitment and subsequent macrophage activation are induced (Fig. 2A). CFD simulations across a range of plaque slopes (5°, 15°, 30°, 45°, and 60°) demonstrate that shear rate and ESS rise markedly toward the middle plaque zone (left, Additional file 1: Fig. S3A). Based on these results, 5° and 60° were designated as representative gentle and steep plaque slopes, respectively, in the microfluidic plaque-on-a-chip model. During macrophage activation, the fusion potential is either inherently elevated or further enhanced using a fusogenic inducer (right, Additional file 1: Fig. S3A). This principle enables the generation of fusogenic macrophage (FM)-derived vesicles as diagnostic tools for targeting vulnerable plaques. The plaque-on-a-chip platform was developed as a microfluidic system in which an endothelial cell (EC, green) monolayer was stabilized after 6 h of culture, followed by perfusion of monocytes (cyan) for 1 day (Fig. 2B). ECs were stained with DAPI (blue) and DiO (green) to distinguish them from monocytes labeled with Dil (red, Fig. 2C).

The co-culture of these cells with steep (60°) plaque slopes results in significantly greater monocyte recruitment onto ECs compared to the gentle (5°) slope. This observation is validated by molecular signatures, as ECs in steep plaques show increased ICAM-1 expression to facilitate monocyte adhesion, while monocytes exhibit increased PIEZO-1 expression in response to hemodynamic disturbance, compared to those in gentle plaques (Fig. 2D). To examine the direct response of monocytes to flow disturbance under varying plaque slopes, an EC-free chip was utilized (Fig. 2E). Monocytes adhering to the steep plaque surface display elongated spindle-like morphologies, as indicated by decreased circularity compared to those on the gentle slope after 1-day of culture (Fig. 2F, Additional file 1: Fig. S3B). This morphological activation is further supported by elevated expression of macrophage activation markers (TNF-α, IL-1β, IL-12) and fusion-related markers (MMP-9, E-cadherin, CD44, and DC-STAMP) in macrophages cultured on the steep plaque compared to the gentle one (Fig. 2G, H). Thus, when steep slopes of plaque elevate ESS and ESSG, macrophages are recruited to the plaque and activated. This situation triggers the membrane fusion mechanism with FM-vesicles through a coordinated set of molecular changes.

### FM-vesicle to detect fusogenic macrophages by self-targeting

**Fig. 3:**
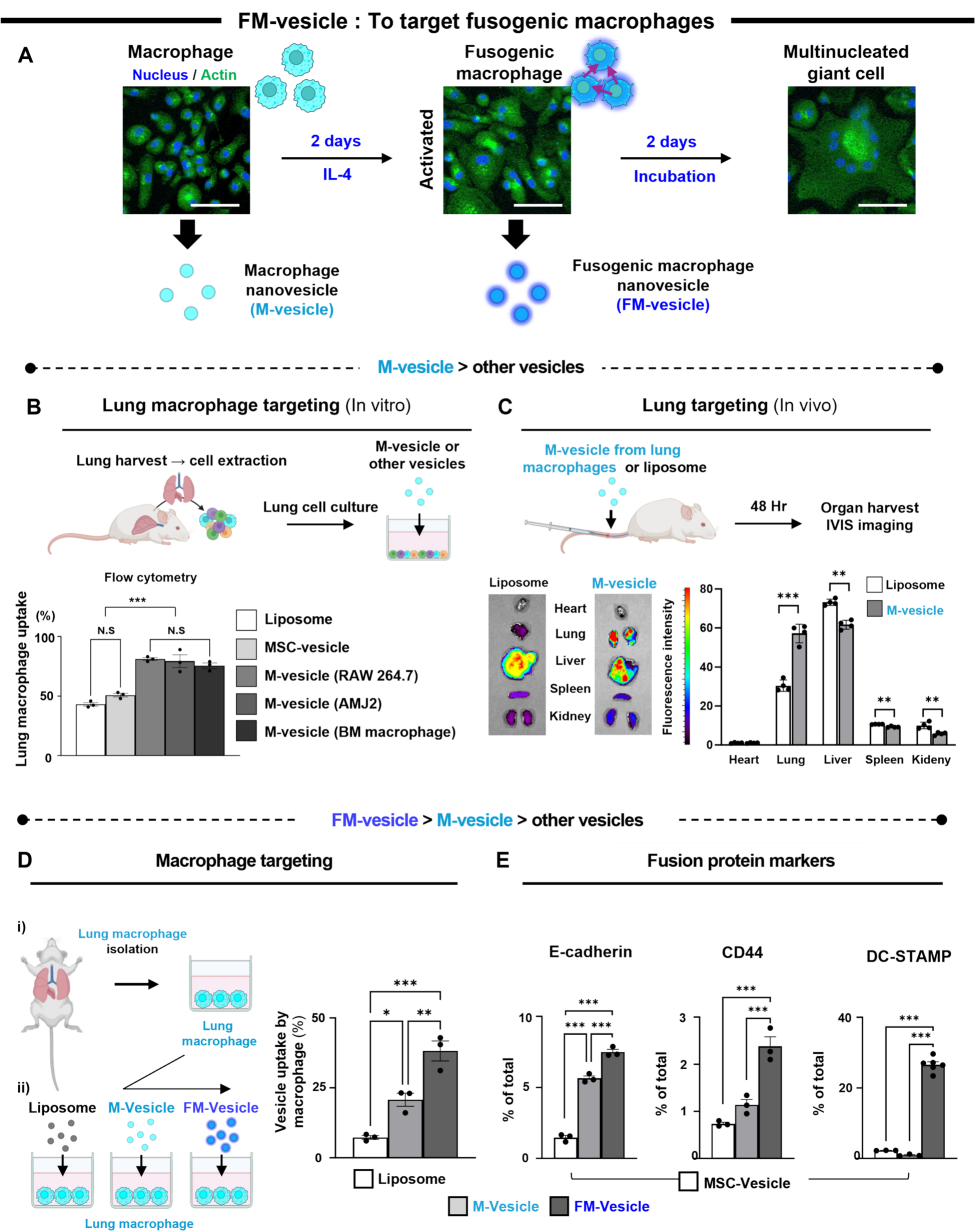
FM-vesicle to detect fusogenic macrophages by self-targeting. **A** Bone marrow (BM)-derived macrophages were isolated from mouse femurs and tibias and cultured under M-CSF treatment for 6 days. Macrophage (M)-and fusogenic macrophage (FM)-vesicles were then produced before and after treating BM-macrophages with IL-4, a fusion inducer, for 2 days, followed by serial filtration. Fusion activation induced by the 2-day IL-4 treatment was confirmed by actin (green) merging and shortened internuclear (blue) distances. Continued culture of fusogenic macrophages for an additional 2 days without NV production resulted in multinucleated giant cells (Scale bars = 50 μm). **B** Mouse lung cells were isolated and cultured without pre-selection by cell type, followed by a 1-h treatment with DiO labeled nano candidates. Uptake of nano candidates (DiO-positive) by macrophages (CD 64-positive) was analyzed via flow cytometry. Lung macrophages preferentially internalized M-vesicles regardless of mother cell origin (RAW 264.7, AMJ2, or BM macrophages), compared to liposomes and mesenchymal stem cell (MSC)-vesicles. **C** Liposomes and M-vesicles derived from lung macrophages were labeled with DiO and intravenously injected into mice for 48 h. IVIS imaging of harvested organs revealed dominant accumulation of both nano candidates in the lung and liver; however, liposomes localized more strongly to the liver, while M-vesicles accumulated preferentially in the lung, where resident macrophages are abundant. This confirms the self-targeting potential of M-vesicles toward their origin cell type. **D** *(i)* Lung macrophages were isolated from mice and cultured to produce M- and FM-vesicles before and after 2-day IL-4 treatment, respectively. *(ii)* After culturing CD64-positive lung macrophages for 1 day, DiO-labeled liposomes, M-, and FM-vesicles were applied for 1 h and analyzed by flow cytometry. Uptake increased sequentially from liposomes to M-vesicles, and further to FM-vesicles. **E** The enhanced fusion potential of macrophages serves as the key mechanistic driver of preferential FM-vesicle uptake. To enable detection by flow cytometry, MSC-, M-, and FM-vesicles were coated with 4 μm beads. Upon treatment, lung macrophages exhibited progressively increased expression of fusion-related proteins (E-cadherin, CD44, DC-STAMP) from MSC- to M-, and further to FM-vesicles, consistent with the uptake pattern. Data are presented as mean ± standard deviation (n: dots represent independent biological replicates in each group). Statistical significance was determined through a two-sided t-test without adjustment for multiple comparisons, as well as one-way ANOVA with Tukey’s test for comparison of lined groups (*p < 0.05, **p < 0.01 and ***p < 0.001)

Macrophages can be activated to form multinucleated cells through cell–cell fusion [29, 30], resulting in enhanced phagocytic capacity and more potent defense functions. Since IL-4 induces macrophage fusion [31], self-targeting to fusogenic macrophages is pursued by producing FM-vesicles. First, bone marrow (BM)-derived macrophages are isolated from mouse femurs and tibias, followed by culture under with macrophage colony-stimulating factor (M-CSF) treatment for 6 days (Fig. 3A). Macrophage (M)-and FM-vesicles are then produced before and after treatment of BM-macrophages with IL-4 as a fusion inducer for 2 days, followed by serial filtration. Fusion activation after the 2-day IL-4 treatment is indicated by actin (green) merging with shortening of inter-nuclei (blue) distances. This process leads to the formation of multinucleated giant cells when fusogenic macrophages are further cultured for an addition 2 more days without nanovesicle (NV) production.

Notably, in the initial activation step (Additional file 1: Fig. S4A), BM-derived monocytes are treated with M-CSF for 6 days to produce macrophages, as indicated by their progressive attachment and spreading. Subsequently, macrophages are treated with IL-4 from day 6 to day 11 to induce fusogenic transformation, which is accompanied by decreased attachment as fusogenic macrophages undergo cell death to complete the activation process. After the 6-day M-CSF treatment to generate macrophages through monocyte attachment (Additional file 1: Fig. S4B), the optimal IL-4 concentration and treatment period are determined by varying the concentration from 0 to 125 ng mL^−1^ until day 11 to effectively produce fusogenic macrophages. As an indicator of optimal fusion before overactivation, the time point immediately preceding the exponential increase in multinucleated giant cells is identified as day 8. Hence, the protocol is optimized by treating cells with a minimum of 50 ng mL^−1^ of IL-4 for 2 days, as this condition yields equivalent marker expression to higher concentrations on day 8.

FM-vesicles exhibit typical spherical morphology (left, TEM image) with an average diameter of 100 nm diameter (middle, DLS) and a surface charge of -42.51 mV (right, zeta potential) (Additional file 1: Fig. S5). Mouse lung cells were isolated and cultured without selection for specific cell types, followed by a 1-h treatment with DiO labeled (green) nano candidates (Fig. 3B). Uptake of the nano candidates (DiO-positive) by macrophages (CD 64-positive) was analyzed via flow cytometry. Lung macrophages preferentially internalized M-vesicles regardless of the mother cell origin (RAW 264.7, AMJ2, and BM macrophage), compared to liposomes or mesenchymal stem cell (MSC)-vesicles. Liposomes and M-vesicles derived from lung macrophages were DIO-labeled (green) and intravenously injected into mice, followed by imaging 48 h later (Fig. 3C). Organ imaging using IVIS revealed dominant accumulation of both nano candidates in the lung and liver, with liposomes showing higher liver accumulation. In contrast, M-vesicles accumulated significantly more in the lung, where resident lung macrophages are located, demonstrating their self-targeting potential toward the cell of origin.

Lung macrophages were isolated from mice and cultured to produce M- and FM-vesicles before and after a 2-day IL-4 treatment, respectively (Fig. 3D). The macrophages (CD64-positive) were then cultured for 1 day and treated for 1-h with DiO-labeled liposomes, M-vesicles, or FM-vesicles to compare self-targeting efficiency via flow cytometry. Uptake increased progressively from liposomes to M-vesicles and further to FM-vesicles, indicating that the fusion potential of macrophages is a key mechanistic factor enhancing the internalization of FM-vesicles (Fig. 3E). To further evaluate fusion-related responses, MSC-, M-, and FM- vesicles were coated with 4 μm beads to increase individual particle size for flow cytometric detection. Treatment with these vesicle types led to a significant increase in expression of fusion protein markers (E-cadherin, CD44, and DC-STAMP) in lung macrophages from MSC- to M- and then to FM-vesicles, which is consistent with the observed uptake patterns.

### Membrane fusion as key of uptake and endosomal escape

**Fig. 4:**
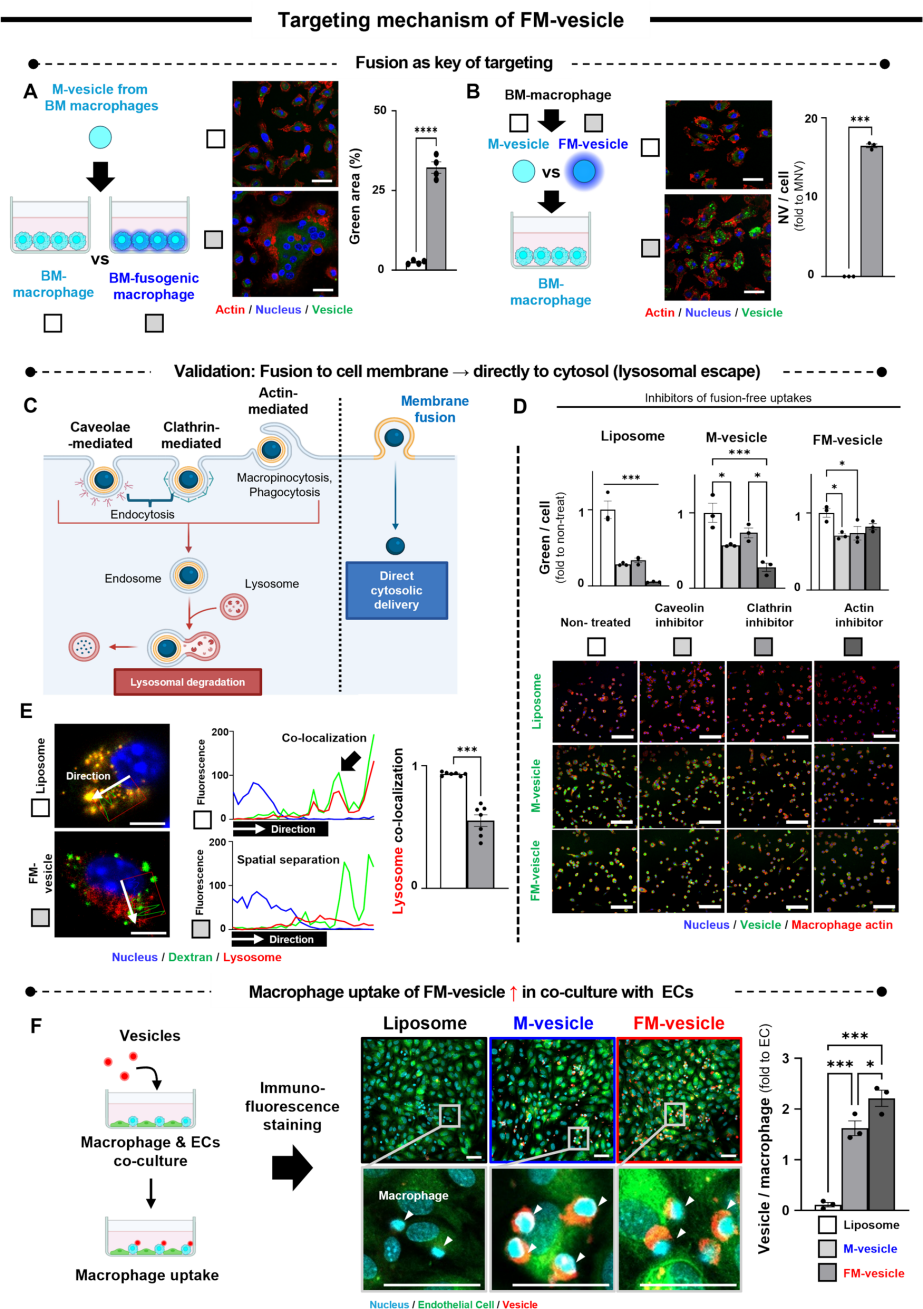
Membrane fusion potential as a key mediator of uptake and endosomal escape in macrophages. From this point onward, BM-macrophages were used to produce M-and FM-vesicles, in addition to generating fusogenic macrophages through a 1-h IL-4 treatment. **A** The elevated membrane fusion potential enables fusogenic macrophages (red actin and blue nuclei) to internalize M-vesicles (green) significantly more efficiently than non-fusogenic macrophages (red and blue, scale bars = 20 μm). **B** Conversely, non-fusogenic macrophages (red and blue) exhibit significantly higher uptake of FM-vesicles (green) compared to M-vesicles (green, scale bars = 100 μm). Together, these findings indicate that membrane fusion potential is a key determinant of vesicle uptake by macrophages. **C** Membrane fusion between vesicles and the cell membrane provides an alternative route to bypass lysosomal degradation via direct delivery to the cytosol. In contrast, endosomal internalization mechanism is mediated by caveolae, clathrin, and actin, typically resulting in degradation and exocytosis through the endo-lysosomal pathway. **D** Each endosomal pathway was inhibited in BM-macrophages (red and blue) for 30 min, followed by overnight incubation with liposomes, M- and FM-vesicles (DiO green). Although uptake of all three nanocarriers decreased, FM vesicles were least affected by inhibition, supporting the involvement of a direct fusion mechanism (Scale bars = 100 μm). **E** Liposomes and FM-vesicles were loaded with hydrophilic dextran (green) to track co-localization with lysosomes (red) in macrophages (blue). Whereas liposomes predominantly co-localized with lysosomes, a significantly greater fraction of FM-vesicles escaped lysosomal association (Scale bars = 5 μm). **F** In co-culture with ECs (vWF-green and blue nuclei), macrophage-specific uptake (blue nucleus indicated by white arrows) after a 1-h treatment with nano candidates (25 μg mL⁻^1^, DiD-red) increased progressively from liposomes to M-vesicles and further to FM-vesicles (Scale bars = 50 μm). Data are presented as mean ± standard deviation (n: dots represent independent biological replicates in each group). Statistical significance was determined through a two-sided t-test without adjustment for multiple comparisons, as well as one-way ANOVA with Tukey’s test for comparison of lined groups (*p < 0.05, **p < 0.01 and ***p < 0.001)

For subsequent experiments, BM-macrophages were used to produce M-and FM-vesicles, as well as fusogenic macrophages, with a 1-h IL-4 treatment. The high membrane fusion potential of fusogenic macrophages drives significantly higher uptake of M-vesicles (green) compared to non-fusogenic macrophages (Fig. 4A). Similarly, non-fusogenic macrophages exhibit significantly greater uptake of FM-vesicles (green) compared to M-vesicles (green) (Fig. 4B). Together, these results demonstrate that membrane fusion potential is a key mediator of vesicle internalization by macrophages. Cells internalize nanoparticles through multiple mechanisms, including caveolin- or clathrin-mediated endocytosis, actin-mediated pinocytosis/phagocytosis, and direct membrane fusion [32–34]. Among these, vesicle-cell membrane fusion provides a mechanism for endosomal escape, delivering vesicles directly to the cytosol, thereby bypassing lysosomal degradation (Fig. 4C). In contrast, caveolae, clathrin, and actin-mediated endocytosis pathways result in vesicle trafficking through the endo-lysosomal pathway, where degradation or exocytosis limits intracellular retention.

Each endosomal internalization pathway was inhibited in BM-macrophages (red and blue) for 30 min (Fig. 4D), followed by overnight internalization of liposomes, M-vesicles, and FM-vesicles (DiO green). Although the internalization of all three nano candidates was significantly reduced, FM-vesicles were the least affected by these inhibitors, highlighting the dominant role of the direct fusion mechanism. Liposomes and FM-vesicles were loaded with hydrophilic dextran (green) to track co-localization with lysosomes (red) in macrophages (blue) (Fig. 4E). In contrast to the dominant co-localization of liposomes with lysosomes, a significantly greater portion of FM-vesicles escaped lysosomal colocalization. In co-culture with ECs (vWF-green and blue nuclei, Fig. 4F), macrophage-specific uptake (blue nuclei indicated by white arrows) of nano candidates after 1-h treatment (25 μg mL⁻^1^, DiD-red) increased progressively from liposomes to M-vesicles and further to FM-vesicles.

### FM-vesicle as a sensor of plaque slope in rabbit carotid arteries

**Fig. 5:**
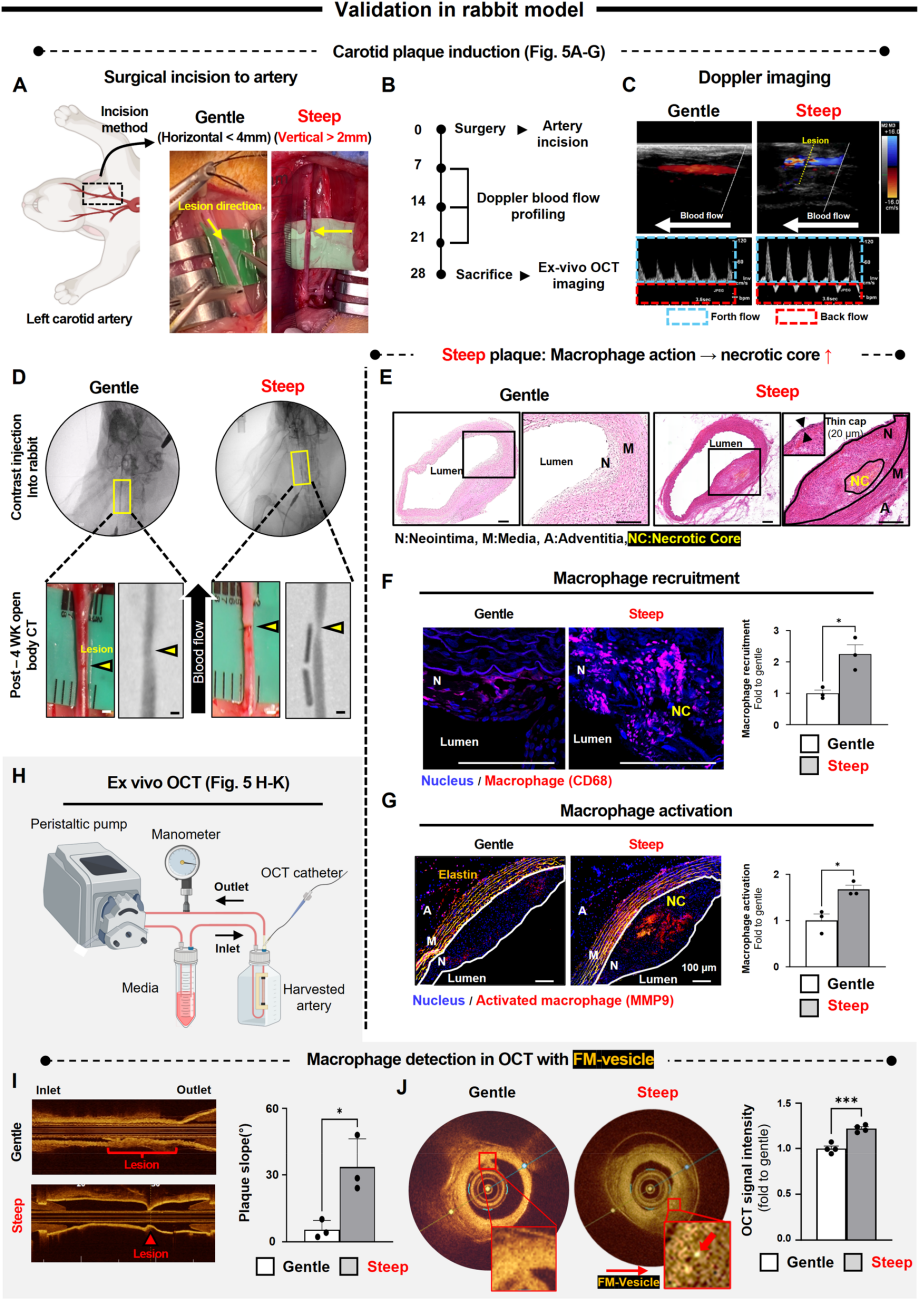
FM-vesicle as a sensor of plaque slope by targeting activated macrophages in rabbit carotid arteries. **A** Gentle and steep plaques were generated by incising rabbit carotid arteries to < 4 mm along the horizontal axis for gentle plaques and > 2 mm along the vertical axis, respectively. **B** Doppler imaging was conducted weekly for three sessions, followed by sacrifice on day 28 and ex vivo optical coherence tomography (OCT) imaging of the harvested arteries. **C** When the plaque slope became steep compared to the gentle one, both forward (blue) and backward (red) flow peaks increased markedly, reflecting disturbed hemodynamics under doppler imaging on day 21. **D** By day 28, angiography revealed visibly greater accumulation of imaging contrast within steep plaques, consistent with a narrower flow channel observed in open-body CT compared to gentle plaques (Scale bars = 1 mm). The arteries were then harvested for the following experiments. **E** H&E staining of plaque regions showed that the steep slope induced a thickened neointima with the formation of a fibrous cap and necrotic core, in contrast to the mild intimal thickening observed with the gentle slope (Scale bars = 200 μm). **F** Immunofluorescence staining demonstrated a significantly higher abundance of macrophages (red CD68) among nuclei (blue) in steep plaques compared to gentle ones. **(G)** Consistently, MMP9 expression (red) was significantly upregulated in steep plaques, indicating macrophage activation in response to disturbed flow (scale bars = 100 μm). **H** The harvested arteries were connected to an ex vivo perfusion system with a peristaltic pump under pressure control. FM-vesicles loaded with gold nanoparticles were introduced to detect activated macrophages within plaques via catheter-based OCT imaging. **I** Cross-sectional OCT images clearly visualized arterial geometries, enabling quantitative analysis of gentle and steep slopes.** J** Perfusion with gold-loaded FM-vesicles yielded enhanced OCT signal intensity in steep plaques compared to gentle ones, reflecting increased accumulation of fusogenic macrophages which was validated by quantitative analysis. Data are presented as mean ± standard deviation (n: dots represent independent biological replicates in each group). Statistical significance was determined using a two-sided unpaired t-test for comparison between the two groups (*p < 0.05, and ***p < 0.001)

Gentle and steep plaques were generated by incising rabbit carotid arteries to < 4 mm horizontally (parallel to the blood flow) for gentle plaques and > 2 mm vertically (perpendicular to the blood flow), respectively (Fig. 5A).

Doppler imaging was performed weekly for three sessions, followed by sacrifice on day 28 and ex vivo OCT imaging of the arteries (Fig. 5B). Compared to gentle plaques, steep plaques (Fig. 5C) exhibited substantially higher forward (blue) and backward (red) flow peaks on day 21, reflecting hemodynamic disturbance. By day 28, angiography (Fig. 5D) showed that steep plaques accumulated the imaging contrast more prominently, corresponding to narrower flow channels observed in open-body CT compared to gentle plaques. The arteries were then harvested for subsequent experiments.

In rabbit carotid arteries after 28 days of surgical incision (Fig. 5E, S6A), H&E staining of steep plaques revealed pronounced thickening of the neointima with the formation of a fibrous cap and necrotic core, in contrast to the mild intimal thickening observed in gentle plaques, as confirmed by quantitative analysis. Immunofluorescence staining further demonstrated that steep plaques contained significantly more macrophages (red CD68) among the cells (blue nuclei) compared to gentle plaques (Fig. 5F). This finding was supported by a significant increase in MMP9 expression (red) in steep plaques (Fig. 5G), corroborated by immunostaining with quantitative analysis (Additional file 1: Fig. S6B), indicating macrophage activation in response to flow disturbance. Moreover, steep plaques exhibited higher vulnerability to stenotic progression compared to gentle plaques, as indicated by increased expression of pro-inflammatory marker genes (TNF-α, IL-1β, IL-12) in PCR analysis (Additional file 1: Fig. S7A). Consequently, more macrophages became fusogenic in steep plaques relative to gentle plaques, as validated by the expression of fusion-related markers (MMP9, E-cadherin, CD44, DC-STAMP) (Additional file 1: Fig. S7B).

The arteries were subjected to an ex vivo perfusion system with a peristaltic pump under controlled pressure (Fig. 5H). FM-vesicles were loaded with gold nanoparticles (30 nm diameter) to detect activated macrophages in the plaques via local surface plasmon resonance [35]. The gold signals were preserved after loading into FM-vesicles, as confirmed by TEM imaging (Additional file 1: Fig. S9A). Upon perfusion through catheter insertion for OCT imaging (Fig. 5I), cross-sectional images clearly visualized the artery geometries, enabling calculation of gentle and steep plaque slopes through image analysis. The distal reference lumen area (yellow), minimal lumen area (red), and lumen stenosis showed no significant difference between the gentle and steep plaque groups (Additional file 1: Fig. S8). Perfusion with gold-loaded FM-vesicles produced brighter OCT signals in steep plaques, indicating greater accumulation of fusogenic macrophages compared to gentle plaques, as confirmed by quantitative analysis (Fig. 5J). Further validation of the gold-based OCT signal was performed using IVIS imaging of the rabbit carotid arteries (Additional file 1: Fig. S9B). Steep plaques exhibited higher signal intensity due to targeted FM-vesicle accumulation with DiD labeling compared to gentle plaques, which was also confirmed quantitatively.

## Discussion

Pathological remodeling of arterial walls occurs through a cascade of abnormalities, beginning with changes in vessel geometry, followed by hemodynamic disturbance, and further progressing to endothelial dysfunction, resulting in inflammatory responses leading to plaque formation. During the initial clinical visit, early detection of plaque formation and identification of progression factors are critical for reducing morbidity and mortality. Despite continued advances in diagnostic modalities such as FFR, OCT, and CT, the recurrence of emergent follow-ups after initially unremarkable findings underscores an unmet need to refine current diagnostic paradigms. The first step of this study was motivated by identifying this diagnostic gap through retrospective analysis of clinical data from 180 patients collected over 9 years, with an average follow-up of 2-years. After 1:1 matching between patients with progressive plaques and healthy controls, the final cohorts were reduced to n = 10 per group. Although the sample size was limited, the data clearly established that steep plaque slopes act as a geometric driver of plaque progression, often associated with emergent clinical events. This finding provided the rationale for focusing on the fusogenic potential of macrophages as a self-targeting mechanism, enabling the development of macrophage-derived nanovesicles as a promising OCT contrast agent for detecting vulnerable plaques.

Single-frame analysis during ongoing OCT examinations often fails to capture unforeseen plaque progression. A key reason, as defined in this study, is that such a simplified approach cannot account for the regulatory influence of patient-specific vascular geometry and the resulting alterations in shear stress, both of which critically drive plaque destabilization. The CFD analysis of hemodynamic parameters required the reconstruction of 3D luminal geometry using 100–200 image frames. However, the conventional OCT and CT could provide only single 2D images, which are far insufficient to generate the 3D luminal geometry. Thus, direct CFD comparison of plaque slope, peak endothelial shear stress (ESS), and steep spatial gradients (ESSG) between multi-frame and single-image frame analyses was technically infeasible. Therefore, the analytical advantage of this study lies in reconstructing vessel geometries from 100 to 200 OCT image frames per patient and applying computational fluid dynamics (CFD) to quantify corresponding changes in hemodynamic parameters. This multidimensional approach significantly enhances the reliability and accuracy of clinical OCT analyses.

In parallel, FM-vesicles represent a technical highlight of this study, aligning with the emerging trend of immune-targeted imaging for identifying high-risk plaque progression (16). This approach is particularly meaningful because it translates the cellular mechanism of monocyte-to-macrophage fusion (where monocytes transition into multinucleated, highly fusogenic cells) into a nanotechnological application, rather than treating macrophages merely as static immune markers. Abnormal hemodynamic changes (e.g. disturbed flow and elevated of ESS and ESSG) induce dysfunctional activation of ECs that increases their permeability, resulting in the infiltration and accumulation of circulating lipids within the vascular wall. The activated ECs release pro-inflammatory cytokines (e.g. TNF-α, IL-1β, and IL-12) to recruit monocytes, which become macrophages upon adhesion [36]. The macrophages transmigrate into the vascular wall with the aid of increased EC permeability and uptake accumulated lipids to form foam cells, while inflammatory cytokines further promote macrophage fusion to produce multinucleated giant cells [37]. Likewise, FM-vesicles were generated by inducing fusogenic macrophages through IL-4 treatment, which also triggers VCAM-1 expression in endothelial cells [38], thereby facilitating monocyte and macrophage adhesion and migration to inflammatory sites [39, 40].

The fusion-related phenotype of macrophages in steep plaques was mediated by PIEZO-1-associated mechanosensitive signaling rather than endogenous IL-4, as evidenced by the lack of IL-4 upregulation in steep plaques (data not shown). IL-4 treatment was used as an experimental condition to produce fusogenic macrophages regardless of the plaque slope (Fig. 3). Steep plaques trigger pathological hemodynamic events, which become the strategic foundation for self-targeting of fusogenic macrophages by FM-vesicles through fusion uptake. Persistent increases of ESS and ESSG activate ECs to express PIEZO-1, which propagate pro-inflammatory signals into the subintimal space. Consequently, expression of fusogenic membrane markers (e.g. MMP-9, DC-STAMP, CD44, and E-cadherin) is upregulated in macrophages (Fig. 2H). Because the same marker profile is enriched in FM-vesicle membranes (Fig. 3E), fusogenic macrophages internalize FM-vesicles through membrane fusion. This mechanistic cascade serves as a key mechanism to specify and enhance OCT signals from steep plaques using gold nanoparticle-loaded FM-vesicles (Fig. 5J). Furthermore, FM-vesicles possess a distinct advantage in evading endosomal degradation through direct cytosolic internalization via membrane fusion, thereby prolonging their intracellular detection function within target macrophages.

As a validation model, the plaque-on-a-chip microfluidic system enables precise recapitulation of plaque slopes to perform user-defined experiments on monocyte recruitment, macrophage fusion, and inflammatory activation. This approach provides a meaningful bridge between cell-based and animal studies, effectively validating the impact of slope geometry on vascular inflammation. However, future applications should integrate additional vascular components such as SMCs, vessel geometry, and shear factors, either in combination or as decoupled controls under real-time monitoring. In parallel, the rabbit carotid ligation model, refined through controlled incision direction to produce gentle and steep plaques, offers an adaptable in vivo platform for studying vascular wall remodeling. This approach was intended to control the slope elevation and protrusion pattern of induced plaque, rather than to reproduce vascular remodeling through the molecular pathogenesis of neointima formation in humans. The rationale for modifying the incision direction was further supported by surgical evidence demonstrating that arteriotomy orientation affects luminal narrowing and postoperative stenosis [41]. Notably, this model enables plaque generation within one month, compared to the one-year duration required for high-fat diet-induced plaques in apolipoprotein E knock-out mice. Moreover, serial ex vivo OCT imaging allows time-course monitoring of plaque morphology, highlighting its utility for investigating the dynamic progression of vulnerable plaques. Nonetheless, direct in vivo imaging results are not presented due to limitations in probe size and system compatibility with the rabbit model, which should be addressed in future studies.

While fractional flow reserve (FFR) remains the clinical standard for functional assessment of coronary lesions, its predictive reliability is limited in identifying plaques that will evolve into vulnerable, rupture-prone states. Accordingly, this study introduces quantitative analysis of plaque slope and associated hemodynamic changes to complement and enhance FFR-based diagnostics. Recent reports showing that non-ischemic plaques with vulnerable morphology can still trigger major adverse cardiovascular events [42, 43] further underscore the need for additional risk markers beyond FFR. By combining patient-specific vessel reconstruction, CFD-based hemodynamic analyses, and OCT imaging enhanced by autologous FM-vesicles, this study proposes a renewed paradigm for imaging-based diagnostics. Through this integrated approach, emergent follow-ups may be minimized, contributing to sustained reductions in morbidity and mortality associated with the world’s leading cardiovascular diseases.

As a point of further study, larger multicenter cohort studies with extended observation periods are necessary, as the current clinical dataset includes a relatively small number of patients in propensity-matched groups with a 2-year follow-up. Although OCT detection of macrophage markers is enhanced by the self-targeting capability of FM-vesicles, imaging artifacts can arise in heavily calcified lesions, reducing the sensitivity and specificity of quantitative analysis an issue that must be addressed for successful clinical translation. In biodistribution studies, a substantial number of macrophage-derived vesicles accumulated in the liver (Fig. 3C), indicating potential hepatic toxicity. When these vesicles were applied to macrophages and ECs in vitro (Fig. 4), no apparent cytotoxicity was observed in both cell types. Nonetheless, more in-depth examination regarding hepatic safety associated with vesicle accumulation is required in the next study for reliable clinical translation. On the other hand, partial hepatic sequestration of FM-vesicles also raises concerns about off-target uptake. Therefore, optimizing the vesicle dosage and incorporating surface modifications to prolong systemic circulation will be critical for improving target specificity.

Notably, whereas the FM-vesicles were derived from mouse bone marrow macrophages, the actual target was rabbit macrophages in the ex vivo model. The membrane fusion mechanism underlying self-targeting by FM-vesicle is conserved across mammalian lineages as indicated by the shared expression of DC-STAMP, CD44, and E-cadherin. However, potential differences in receptor binding affinity, membrane composition, and fusogenic protein homology between the two species may still influence detection efficiency. Indeed, mouse macrophages lack the expression of the VLDL receptor protein, which is readily detectable in rabbit and human macrophages [44]. Surface markers such as F4/80 and RAM-11 are used to identify murine and rabbit macrophages, respectively, which may also may limit cross-species applicability [45, 46]. These facts underscore substantial differences in membrane protein composition between the two species. Future studies should utilize species-matched FM-vesicles to validate targeting specificity and to analyze detection efficiency prior to clinical translation.

The rabbit model effectively reproduces plaque slope–induced flow disturbances and macrophage recruitment; however, future studies should further model the chronic inflammatory milieu and systemic risk factors characteristic of human atherosclerosis. Finally, technical constraints in OCT imaging systems must be overcome to enable real-time intravascular imaging in live animals that fully replicate native vascular conditions prior to clinical application. Current intravascular catheters of clinical-grade OCT systems are fundamentally designed for human coronary anatomy. Thus, only catheters with outer diameters of 4–8 French (1.35–2.7 mm) are available. In contrast, the rabbit coronary artery has a luminal diameter of approximately sub-millimeter to 1 mm [47], far below the 3–5 mm range of the proximal left anterior descending, left circumflex artery, and right coronary artery in humans [48,49]. This anatomical mismatch represents a fundamental barrier to direct intravascular OCT imaging in the rabbit model. Inserting an OCT probe into the rabbit carotid artery poses generates risks of luminal occlusion, mechanical injury to the vessel wall, and incomplete catheter apposition. Each risk compromises imaging fidelity and hemodynamic measurements. Furthermore, commercially available OCT pullback systems are calibrated for human coronary flow rates and vessel geometry, making direct application to rabbit models technically unreliable without dedicated hardware modification.

To address these constraints while preserving physiological relevance, this study employed an ex vivo pressure-controlled perfusion system as a replacement for the native intravascular environment. Rabbit arterial segments were isolated and maintained under continuous perfusion using a peristaltic pump, and the intraluminal pressure was calibrated to simulate the physiological pressure range of the rabbit carotid artery. This closed-loop perfusion circuit enabled OCT catheter insertion under controlled hemodynamic conditions, free from cardiac motion artifacts and respiratory noise inherent to live animal imaging. Importantly, the measurements of plaque slope using OCT were consistent with Doppler flow profiles and angiographic findings which were obtained in vivo prior to sacrifice, further validating the reliability of the ex vivo model. These technical constraints should be resolved through catheter miniaturization or the development of OCT platforms dedicated to small animals, which remain an inevitable step toward real-time in vivo imaging and clinical translation.

## Conclusion

This study proposes a plaque slope (steep > 15°) as a diagnostic hemodynamic biomarker for evaluating patient-specific vascular geometry, driven by its causative role in macrophage activation under plaque progression. To establish this framework, 3D vessel geometries were reconstructed from 100–200 OCT frames per patient, followed by analyses using computational fluid dynamics (CFD) analysis. This approach refines the current diagnostic paradigm by overcoming the resolution limits of conventional, single-frame OCT analysis. This insight guided the development of FM-vesicles as a molecular imaging agent to enable self-targeting of fusogenic macrophages, which were activated by hemodynamic disturbance due to steep plaque slopes. The improvement in targeting efficiency and accuracy was validated across microfluidic, ex vivo, and in vivo models. Notably, gold nanoparticle-loaded FM-vesicles enhanced the OCT contrast specifically in fusogenic macrophages to diagnose plaque progression with steep slopes. As FM-vesicles can in principle be generated from autologous monocytes, this approach offers a path toward patient-specific imaging without the risk of immune rejection. Together, this study provides a clinically translatable strategy to avoid diagnostic outliers resulting from unexpected plaque progression in coronary artery disease.

## Methods/experimental

### Clinical data analysis

This prospective study was conducted as a part of the Integrated Coronary Multicenter Imaging Registry in South Korea (ClinicalTrials.gov: NCT03298282) across four institutions. Patients presenting with chest pain underwent coronary angiography, followed by either OCT or FFR assessment according to clinical indication. Eligible participants were 20–80 years old and exhibited 50–70% coronary stenosis on angiography. Exclusion criteria included: (i) hypersensitivity to contrast media, (ii) the need for vasopressor support, (iii) left ventricular ejection fraction under 30%, (iv) renal dysfunction (creatinine > 2 mg dL^−1^), (v) non-cardiac life expectancy of less than 1 year, and (vi) critical valvular heart disease.

A total of 180 patients were prospectively enrolled over a 9-year period and underwent coronary FFR measurement, OCT, and CT angiography, with a median follow-up duration of two years (Fig. 1B). Among these, 48 patients were excluded due to missing baseline or follow-up OCT data. Of the remaining patients, 14 demonstrated plaque progression requiring revascularization, with FFR decreasing from > 0.9 to < 0.85 or from > 0.85 to < 0.8. Four of these were excluded, one due to missing OCT imaging of the progressive lesion and three due to in-stent restenosis, yielding a final progression group of 10 patients.

The initial control group comprised 118 patients without major adverse cardiovascular events and with stable FFR during follow-up. Propensity-score matching was applied at a 1:1 ratio, selecting 10 control patients whose baseline characteristics were statistically comparable to those of the progression group. No significant differences were observed between the groups in i) demographic or clinical parameters (age, sex, BMI, hypertension, diabetes, hypercholesterolemia, smoking status; Table 1) or ii) angiographic and OCT measurements (Table 2).

Compared to the matched controls, the plaque progression group exhibited significantly more adverse hemodynamic and morphological features, including steeper plaque slope, higher shear rate, lower ESS, and greater macrophage infiltration (all *p* < 0.001). The study was conducted in accordance with the Declaration of Helsinki, and written informed consent was obtained from all participants. The study protocol was approved by the Institutional Review Board of Yonsei University College of Medicine (IRB No. 1-2017-0049).

### 3D vascular modeling

Patient-specific 3D anatomy of the coronary artery was reconstructed by integrating OCT images with CT coronary angiography (CTCA) data. CTCA segmentation was performed using automated image analysis software (DicomViewer Pro, version 2.11, Inobitec, Voronezh, Russia). Centerlines of the main vessel and side branches were extracted using VMTK (Orobix Srl, Bergamo, Italy).

OCT-based segmentation of the vessel lumen was conducted in MATLAB R2023b (Mathworks, Natick, MA, USA), followed by manual review to ensure accuracy and reproducibility. The centroid of each segmented lumen was aligned to the centerline of the main vessel, with lumen contours oriented perpendicularly. The absolute position and orientation of each OCT frame were determined using side branches as anatomical landmarks. Relative orientations between frames were established using a sequential triangulation algorithm, progressively connecting lumen contours via triangulated meshes to generate a continuous 3D vessel model {Athanasiou, 2017 #53}.

Side branches were incorporated into the final 3D model if they met the established criteria outlined in the Expert Guidelines: i) a diameter greater than 1 mm or 1/3 that of the main vessel, and ii) a bifurcation originating from one vessel longitudinally proximal or distal to the region of interest. The diameter of each side branch was measured from CTCA and adjusted to match the corresponding OCT frames, ensuring consistency across modalities.

Macrophage accumulation was qualitatively assessed on OCT images by identifying the bright, punctate, signal-rich regions exhibiting high backscattering and shadowing. Quantitative analysis was performed by calculating macrophage density over non-overlapping 10-frame segments, defined as the number of macrophage-positive frames per segment. This approach standardized the assessment and reduced local variability in evaluating macrophage burden.

### Boundary conditions and CFD

Arterial geometries were reconstructed and meshed using Ansys Fluent Meshing (ANSYS Inc., Canonsburg, PA, USA). Local mesh resolution was increased via tight refinement to ensure smooth reconstruction across disconnected boundaries between vessel wall frames. Mesh quality was verified for each model by maintaining a skewness below 0.9, ensuring numerical stability and convergence. Blood flow was modeled using the Navier–Stokes equations as a non-Newtonian, incompressible, continuous fluid with a density of 1060 kg m^−3^. Shear rate-dependent viscosity of coronary flow was simulated using the Carreau model to replicate physiologically relevant rheological behavior.

Patient-specific CFD simulations were performed in Ansys Fluent (ANSYS Inc.) by incorporating inlet flow conditions derived from the frame count method of the Thrombolysis in Myocardial Infarction (TIMI). A uniform velocity profile was applied at the inlet, and a 3-cm extension pipe was included to allow fully developed flow prior to entry into the reconstructed artery. Outlet boundary conditions were assigned according to Murray’s law by setting proportional flow rates to the cube of the vessel diameter [50].

Endothelial shear stress (ESS) distributions were mapped at the plaque entry zone to characterize the local hemodynamic environment. ESS values were extracted at identical longitudinal positions along the vessel to allow direct comparison with OCT images. Each luminal cross-section was subdivided into 15° angular bins, and the mean ESS within each bin was calculated to capture circumferential variation. These data were compiled into 2D heatmaps, with x- and y-axes representing angular bins and longitudinal positions, respectively, enabling visualization of circumferential ESS variation along the vessel.

### Cell culture

Primary human umbilical vein ECs (HUVECs, Lonza, C2519A, Basel, Switzerland, passages 4–8) were maintained in endothelial growth medium MV2 (EGM, C-22022, PromoCell, Heidelberg, Germany). RAW 264.7 monocytes (ATCC, TIB-71, VA, USA, passage 42) were cultured in Dulbecco’s Modified Eagle Medium (DMEM, 11995-065, Thermo Fisher, Waltham, MA, USA) supplemented with 10% (v v^−1^) fetal bovine serum (FBS, 16000-044, Gibco, Waltham, MA, USA) and 1% (v v^−1^) penicillin–streptomycin (PS, 15140-122, Gibco) at 37 °C in a humidified atmosphere with 5% CO_2_.

The alveolar macrophage cell line (AMJ2-C11, ATCC, CRL-2465, VA, USA, passages 5–10) and human bone marrow-derived mesenchymal stem cells (MSCs, ATCC, PCS-500-012, VA, USA, passages 6–12) were used in experiments targeting lung macrophages. Under standard culture conditions (37 °C, 5% CO_2_), AMJ2-C11and MSCs were cultured in complete DMEM with 5 mM HEPES [4-(2-hydroxyethyl)-1-piperazineethanesulfonic acid] and low-glucose DMEM (1.5 mg L^−1^), respectively.

### Plaque-on-a-chip in microfluidics

A model of vascular inflammation was produced using a microfluidic system in which HUVECs underwent inflammatory activation by treatment with 10 ng mL^−1^ human TNF-α (300-01A, Thermo Fisher Scientific) for 24 h, followed by perfusion of monocytes. Cell interactions were visualized by pre-labeling of HUVECs and RAW 264.7 monocytes with DiO (green) and DiI (red), respectively, at a 1:500 ratio for 30 min using the Vybrant Multicolor Cell-Labeling Kit (V22889, Thermo Fisher Scientific) on 2D polystyrene culture plates prior to introduction into the microfluidic chip.

The slope geometry characteristic of vascular plaque was recreated by engineering either a gentle 5º slope or a steep 60º slope at the channel center of a polydimethylsiloxane (PDMS) microfluidic chip (35 × 0.6 × 0.6 mm; length × depth × height). HUVECs were seeded at 1 × 10^6^ cells mL^−1^ and allowed to adhere for 6 h. Endothelial growth medium was then perfused at 1076 μL minute^−1^ for 24 h using a peristaltic pump (BT100-1L, Longer Precision Pump Co., Ltd, Baoding, Hebei, China) to mimic physiological shear stress and arterial hemodynamics.

Cells were fixed in 4% paraformaldehyde (PFA; CNP015-0550; CellNest, Hanam-si, Gyeonggi-do, Republic of Korea) for 20 min at room temperature, washed three times with PBS, permeabilized with 0.1% Triton X-100 (T8787, Sigma-Aldrich, Burlington, MA, USA) for 10 min, and blocked with 5% bovine serum albumin (BSA, A0100-005, GenDEPOT, Katy, TX, USA) in PBS for 1 h. Primary antibody staining was performed overnight at 4 °C using ICAM-1 (1:200, ab2213, Abcam, Cambridge, UK) and PIEZO-1 (1:200; MA5-32876, Thermo Fisher Scientific), followed by three PBS washes. Secondary antibody staining was performed for 2 h at room temperature using FITC-AffiniPure goat anti-mouse IgG (1:500; 115-095-003; Jackson ImmunoResearch, West Grove, PA, USA) for ICAM-1 and Alexa Fluor 594-conjugated AffiniPure goat anti-rabbit IgG (1:500, 115-585-003, Jackson ImmunoResearch) for PIEZO-1. After three additional PBS washing, nuclei were counterstained with DAPI. Confocal images were acquired using a Zeiss LSM 900 microscope (Oberkochen, Germany) with Zen 3.3 Blue Edition software, and quantitative analyses were performed in ImageJ (Fiji, version 1.52i, National Institutes of Health, MD, USA).

### Macrophage isolation with fusogenic induction

All animal procedures were approved by the Institutional Animal Care and Use Committee (IACUC) of Yonsei University College of Medicine (#2022-0208 for mouse experiments, #2023-0101 for rabbit experiments). Bone marrow macrophages were isolated from wild-type C57BL/6 mice (male, 6 weeks old, Orient Bio, Seongnam-si, Gyeonggi-do, Republic of Korea). Following CO₂ euthanasia, femora and tibiae were excised, and the epiphyses at the knee ends were removed to expose the metaphyseal region. Bone marrow was extracted by inserting a 20-gauge needle through the bottom of a sterile 0.7 mL microcentrifuge tube placed inside a sterile 1.7 mL collection tube. The marrow was drawn into the collection tube and centrifuged at 10,000 × g for 15 s to pellet marrow cells. The pellet was resuspended in 1 × RBC lysis buffer (420,302. BioLegend, San Diego, CA, USA) and incubated for 3 min with gentle agitation every 30 s. Cells were centrifuged at 700 × g for 3 min, washed in complete DMEM, and filtered through a 70 μm strainer to remove clumps and debris.

Mouse fibroblast L929 cells (1 × 10^5^ cells in a 175 cm^2^ culture flask) were cultured for 14 days, to produce conditioned medium, which was mixed with complete DMEM at 8:2 ratio [51] and supplemented with 10 ng mL^−1^ M-CSF (315–02; PeproTech, Cranbury, NJ, USA). Isolated bone marrow cells were cultured in this medium at 1 × 10^6^ cells mL^−1^ on non-adherent Petri dishes for 6 days to induce fusogenic potential. Thereafter, the medium was replaced with complete DMEM containing 100 ng mL^−1^ murine IL-4 (214-14-100, PeproTech) and 10 ng mL^−1^ M-CSF for an additional 2 days, allowing macrophages to mature into multinucleated giant cells, which served as a source for fusogenic (F) macrophage (M)-vesicles.

### Production and characterization of F/M-vesicles and liposomes

Macrophages were cultured in fusogenic induction medium and gently detached from petri dishes with PBS on day 3 and day 6 as immature and mature macrophages, respectively followed by IL-4 treatment to collect fusogenic macrophages on day 8. Vesicles were produced by centrifuging macrophages (1 × 10^8^ cells mL^−1^) at 700 × g, followed by resuspension of the pellet in hypotonic lysis buffer supplemented with 1 × protease inhibitor cocktail (78,440, Thermo Fisher Scientific) and 1.5 mM MgCl_2_ (M1028, Sigma-Aldrich). The cell suspension was incubated at 4 °C for 20 min to facilitate lysis while preserving the protein integrity. Mechanical extrusion was performed by passing the lysate 80 times through a Mini-Extruder (610,000, Avanti Polar Lipids Inc., Alabaster, AL, USA), followed by centrifugation at 700 × g for 12 min to remove unbroken debris. The supernatant was further centrifuged at 14,000 × g for 40 min to pellet down the vesicles. The vesicle pellet was resuspended in PBS and extruded 13 times through a 0.1 μm membrane filter (800,282, Cytiva, Marlborough, MA, USA) placed between two drain discs (PETEDD9025, Sterlitech Corporation, Auburn, WA, USA), ensuring controlled size distribution and removal of aggregates.

Vesicle concentrations were determined using a BCA protein assay kit (23,227, Thermo Fisher Scientific) based on surface protein content. Gold FM-vesicles were produced by resuspending the M-vesicle pellet in 1 mL of gold nanoparticle suspension (753,629, Sigma-Aldrich) prior to extrusion. The nanoparticle size was set to be 30 nm to achieve maximum signal amplification under OCT. Particles larger than 50 nm exhibited reduced loading efficiency and inner-body stability, whereas particles below 10 nm yielded poor signal intensity. Vesicles were also loaded with a fluorescent tracer by allowing macrophages to internalize FITC-dextran (70 kDa, 46,945, Sigma-Aldrich) overnight, followed by lysis and extrusion to encapsulate the tracer within the vesicle lumen.

Liposomes were prepared from a mixture of dipalmitoyl phosphatidylcholine (850355P, Sigma-Aldrich), cholesterol (C8667, Sigma-Aldrich), and 1,2-distearoyl-sn-glycero-3-phosphoethanolamine-N-[methoxy(polyethylene glycol)] (DSPE-PEG, 880120P, Sigma-Aldrich) at a molar ratio of 55:40:5. Lipids were dissolved in ethanol (E7023, Sigma-Aldrich) at 72 °C and rapidly injected using a 20-gauge needle into an equal volume of PBS under stirring at 500 rpm for 5 min, followed by the addition of × 4 volumes of PBS with continued stirring at room temperature for 15 min to ensure complete liposome formation. Ethanol diffusion promoted spontaneous self-assembly of lipid bilayers by minimizing exposure of hydrophobic tails to the aqueous phase, forming stable liposomal structures. Liposomes were extruded seven times before use, and concentrations were determined based on particle counts in the final suspension. For fluorescence tracing, lipids were injected into an equal volume of FITC-dextran solution to enable encapsulation during vesicle assembly.

Vesicles and liposomes were visualized by transmission electron microscopy (TEM, JEM-2100, JEOL, Tokyo, Japan) after staining with 1% uranyl acetate for 30 s, drying for 1 h, and mounting on Formvar-carbon coated grids. Size distribution and particle concentration were measured using nanoparticle tracking analysis (NTA) on the Nanosight NS300 (Malvern Panalytical, Malvern, UK) with three independent 1-min recordings under microfluidic flow of vesicles diluted in PBS (1:10,000). Zeta potential was analyzed (ELS-Z1000, Otsuka Electronics Ltd., Osaka, Japan) after diluting particles with PBS (1:20,000). Liposomes and vesicles were labeled with Vybrant™ DiO (V22886, Waltham, MA, USA) or Vybrant™ DiD (V22887) by incubating with each Cell Labeling Solution at a 1:150 dilution for 30 min at 37 °C, followed by three PBS washes prior to use.

### Targeting lung macrophages

Whole lung tissues were harvested from mice under CO₂ euthanasia and incubated in 10 mL RPMI 1640 medium (11,875,093, Gibco, Waltham, MA, USA) with 1 mg mL⁻^1^ collagenase type II (LS004176, Worthington Biochemical Corporation, NJ, USA) without FBS for 30 min under gentle shaking. Enzymatic activity was terminated by adding 10 mL of complete RPMI 1640, followed by centrifugation at 700 × g for 3 min. The pellet was resuspended and incubated in 1 mL of 1 × RBC lysis buffer for 3 min with gentle shaking every 30 s, followed by centrifugation at 700 × g for 3 min and resuspension in complete RPMI 1640. The cell suspension was filtered through a 70 μm strainer to obtain single-cell lung preparations. Primary alveolar macrophages were isolated by cannulating the trachea with a 20-gauge catheter and performing lung lavage with 1 mL of chilled PBS, repeated ten times. Lavage fluids were pooled, centrifuged at 700 × g for 5 min, resuspended in complete RPMI 1640 medium, and filtered through a 70 µm cell strainer.

Mouse lung cells or alveolar macrophages were seeded at 1 × 10^6^ cells mL^−1^ in 96-well plates and incubated with 25 µg mL⁻^1^ of FM- or MSC-vesicles from RAW 264.7, AMJ2, or bone marrow, as well as DiO-labeled liposomes (particle number equivalent to vesicles). Cells were then fixed with 4% PFA in PBS for 15 min at RT and washed three times with PBS to remove residual fixative. Immunostaining of immune cells and macrophages was performed by incubation with APC/Cyanine 7 anti-mouse CD45 (1:100, 103,116, BioLegend, San Diego, CA, USA) and PE/Cyanine 7 anti-mouse CD64 (1:100, 139,314, BioLegend), respectively, for 1 h at 4 °C. After washing twice, cells were resuspended in 400 uL PBS containing 0.5% FBS to minimize cell aggregation and non-specific binding, followed by flow cytometric analysis (FACS Verse IIIII, BD Biosciences, Franklin Lakes, NJ, USA). Macrophages were identified by gating on CD45^+^CD64^+^ cells in which the proportion of DiO (vesicle)-positive macrophages was quantified to assess vesicle uptake by macrophages.

In vivo organ targeting was examined in mice by injecting DiD-labeled liposomes and M-vesicles in suspension with 200 µL of saline into the tail veins. After 48 h, mice were euthanized with CO₂, and organs were harvested. Biodistribution analysis was then carried out by quantifying the DiD fluorescence intensity of each organ using an in vivo imaging system (IVIS, 124,262, PerkinElmer, Waltham, MA, USA).

Surface display of fusogens on MSC-, M-, and FM-vesicles were analyzed by bead-assisted flow cytometry. Vesicles (40 µg) were incubated with 1 µL of 4 µm aldehyde/sulfate latex beads (A37304, Thermo Fisher Scientific) in 1 mL PBS on a rocker overnight at RT. The coupling reaction was terminated by incubation with 110 mM glycine (GR1021-100-00, BioSesang, Yongin-si, Gyeonggi-do, Republic of Korea) for 30 min. The suspension was then centrifuged at 14,000 × g for 30 min to pellet down the bead-bound vesicles, followed by two washes with 1 mL PBS containing 0.5% FBS. The pellets were resuspended in 100 uL PBS with 0.5% FBS and incubated with primary antibodies against CD44 (1:100, NBP1-47386, Novus Biologicals, Centennial, CO, USA), E-cadherin (1:100, 3195, Cell Signaling, Danvers, MA, USA), and DC-STAMP (1:100, NBP1-79329, Novus Biologicals) for 30 min at RT. After centrifugation at 14,000 × g for 30 min and washing twice, the pellets were resuspended in PBS with 0.5% FBS and incubated for 30 min at RT with secondary FITC-AffiniPure goat anti-mouse IgG (1:400, Jackson ImmunoResearch) for CD44, Alexa Fluor 488-AffiniPure goat anti-rabbit IgG (1:500, Jackson Immuno Research) for E-cadherin, and Alexa Fluor 488-AffiniPure goat anti-rabbit IgG (1:500,, Jackson Immuno Research) for DC-STAMP. The pellets were washed three more times and resuspended in 400 uL PBS with 0.5% FBS for flow cytometric analysis.

### Fusogenic potential in uptake

The uptake of M-and FM-vesicles was compared between macrophages and fusogenic macrophages. Cells were seeded in 24-well plates with glass-like polymer bottoms (2.5 × 10^5^ cells per well, P24-1.5P, Cellvis, Mountain View, CA, USA). Vesicles (25 µg mL^−1^) were labeled with DiO, dispersed in complete DMEM, and incubated with the cells for 1 h at 37 °C. After incubation, wells were washed twice with PBS to remove unbound vesicles prior to downstream analysis.

The role of fusogenic potential in vesicle uptake was further scrutinized by inhibiting non-fusion mechanisms. Thus, caveolae- and clathrin-mediated endocytoses were inhibited with nystatin (25 μM, N6261-500KU, Sigma-Aldrich) and chlorpromazine hydrochloride (12.5 μM, C8138-5G, Sigma-Aldrich), respectively, in addition to actin polymerization with cytochalasin D (20 μM, 8273, Sigma-Aldrich). FM (2.5 × 10^5^ cells per well) were pre-treated with each of these inhibitors for 30 min, followed by overnight incubation with DiO-labeled particles to compare their uptake by fusogenic macrophages.

Macrophages were treated with TNF-α (10 ng mL^−1^) for 24 h to induce an inflammatory phenotype. For co-culture experiments, macrophages and HUVECs were seeded together at a 1:1 ratio (2.5 × 10^5^ cells per well) in 24-well plates, followed by incubation with DiD-labeled vesicles for 1 h and washing twice with PBS to remove unbound particles. Samples were then fixed with 4% PFA in PBS for 30 min at RT and washed three times with PBS. Permeabilization was performed using 0.2% Triton X-100, and nonspecific binding was blocked with 5% BSA in PBS. Actin cytoskeleton and nuclei were stained with rhodamine phalloidin (1:1000, R415, Thermo Fisher Scientific) and DAPI (R37606, Thermo Fisher Scientific), respectively. Uptake of DiO-vesicles was imaged by confocal microscopy and quantified using ImageJ. Lysosomal colocalization was assessed by preloading liposomes or FM-vesicles with 70 kDa FITC-dextran, followed by immunostaining with PE-CD107a (1:100, 121,612, BioLegend) and DAPI, with confocal imaging. The degree of colocalization was quantified using ImageJ and the JACoP plugin by applying Pearson’s coefficient to refine statistical analysis of signal overlap. In the co-culture, HUVECs were additionally stained with primary anti-vWF (1:200, ab6994, Abcam) and secondary Alexa Fluor 488 (1:500, 111–545-003, Jackson ImmunoResearch) to distinguish endothelial cells and quantify vesicle uptake specifically in macrophages using ImageJ.

### qRT-PCR

Total RNA was extracted from cells after microfluidic culture, vesicle-treated macrophages, and rabbit carotid arteries using TRIzol reagent (15,596,018, Thermo Fisher Scientific). cDNA was synthesized from 1 µg of RNA using AccuPower CycleScript RT Pre-mix (Bioneer, Daejeon, Republic of Korea). Primer sequences were designed with NCBI Primer-BLAST and synthesized by Cosmogenetech (Seoul, Republic of Korea). Quantitative real-time PCR was performed using cDNA, SYBR Green, and gene-specific primers on a StepOne Real-Time PCR system (Applied Biosystems, Waltham, MA, USA) (Table 3). Cycling conditions consisted of initial denaturation at 95 °C for 10 min, followed by 40 cycles of denaturation at 95 °C for 1 min and annealing at 60 °C for 1 min. Gene expression levels were normalized to housekeeping genes (β-actin and GAPDH), and relative expression was calculated using StepOnePlus software version 2.3.

**Table 3:** Primer sequences

Gene	Forward (5′→3′)	Reverse (5′→3′)
GAPDH	GACCACTTCGGCATTGTGGA	ATGCCAGTGAGTTTCCCGTT
TNF-α	CCGTCTCCTACCCGAACAAG	AAGGTCCAGGTACTCAGGCT
IL-1β	TGTCAGTCGTTGTGGCTCTG	TTGCAGAGGACGGGTTCTTC
IL-12	CCACAAAACCCCTCCCTTGA	AGGCATGGGGTCATCCTTCA
MMP-9	CGGAGACGGGTATCCTTTCG	CGGCGTTTCCAAAGTACGTG
E-cadherin	TCGCCTACGAAATCCTCAGC	TCTGCGGGTTGATCCTGAAC
CD44	TGCCTACCATGGCTCAGATG	ACGTGCCCTTCTATGAACCC
DC-STAMP	TGTCCTCCCGCTGAGTAAGA	CCAGAAAGACGGGACGACAA

### Rabbit model of carotid artery plaque

Male New Zealand White rabbits (3 kg, Doo Yeol Biotech, Seoul, Republic of Korea) were acclimated for 7 days in a large-animal care facility before experimentation. Anesthesia was induced via intramuscular injection of Zoletil™ (10 mg kg^−1^, Virbac Korea, Seoul, Republic of Korea) and maintained with endotracheal inhalation of isofluorane (2.2%, Hana Pharm, Seoul, Republic of Korea). For plaque formation, a midline incision was made on the anterior neck to expose the left carotid artery, followed by careful dissection of subcutaneous tissue and neck muscles. Bypass branches were ligated with ST-B-1 V micro vessel clamps (8 mm, 00396, Stark Medical Pty Ltd, Artarmon, Australia) to prevent blood leakage. Both proximal and distal ends of the left carotid artery were secured with 6–0 black silk sutures, and a 2 mm inner-diameter silicone tube with a pre-threaded line was inserted to occlude the artery, generating either gentle or steep plaque geometries.

An incision was made in the carotid artery and immediately ligated using 9–0 Ethilon sutures, with the incision size and direction controlled at < 4 mm horizontally (parallel to the blood flow) for gentle plaques and > 2 mm vertically (perpendicular to the blood flow) for steep plaques. Proper occlusion and hemostasis were confirmed before removal of the silicone tube. Soft tissues were closed with 4–0 Vicryl sutures, and the skin was closed with 4–0 Ethilon sutures. Postoperative recovery was monitored daily for 30 days, with no complications observed. Pain was managed through the oral intake of meloxicam (Medica Korea, Seoul, Republic of Korea) at 0.5 mg kg^−1^ daily for 7 days. Infection prophylaxis was maintained through oral intake of enrofloxacin (CTCBio Inc., Hwaseong-si, Gyeonggi-do, Republic of Korea) at 10 mg kg^−1^ once daily for the first 7 days post-surgery. The injury-induced plaque model was developed in rabbit carotid arteries based on our previous observation that vessel injury contributed to neointima formation [52]. This concept is further supported by experimental studies showing that surgical or perivascular manipulation of arteries can induce neointimal hyperplasia or atherosclerotic lesion formation [47, 48].

### Doppler sonography and X-ray angiography

Blood flow of the left carotid artery was monitored after surgery by ultrasonography (iU22 xMatrix DS, Philips, Amsterdam, Netherlands) on days 0, 7, 14, 21, and 28. Both color Doppler and pulse-wave Doppler modes were used to quantitatively assess vascular flow dynamics. Angiography was performed using a C-arm X-ray system (General Electric, Boston, MA, USA). The anterior midline of the neck was incised to expose the artery, and a proximal puncture site was created at least 2 cm from the incision using a flexible catheter needle for central venous access. A 4-French catheter was inserted into the artery and a 1:1 mixture of normal saline and contrast agent (Scalnux, SANOCHEMIA, Neufeld an der Leitha, Austria) was administered to opacify the lumen and obtain high-resolution angiographic images.

### Ex vivo OCT

A perfusion device was 3D-printed (Saturn 4 Ultra, Elegoo, Shenzhen, China) with internal widths of 45, 50, 60, and 70 mm, a depth of 20 mm, and a height of 30 mm to accommodate rabbit carotid artery segments of varying lengths. The device featured a 1.6-mm diameter luer connected with silicone tubing (T23-140-004, iXAK, Seoungdon-gu, Seoul, Republic of Korea) to enable controlled ex vivo perfusion of vessel segments.

On day 28 post-surgery, rabbits were anesthetized via intramuscular injection of Zoletil (50 mg kg^−1^) and xylazine (5 mg kg^−1^). The carotid ligation site was surgically reopened, and both the distal and proximal ends of the artery were ligated with 6–0 black silk sutures. The isolated arterial segment was positioned centrally within the customized perfusion device. Euthanasia was performed by intravenous injection of potassium chloride (20 mg kg^−1^, Choongwae Pharma Corporation, Gwacheon-si, Gyeonggi-do, Republic of Korea). The perfusion device was connected to a manometer (DPG3000, Golden Mountain Enterprise Co., Ltd., Kaohsiung, Taiwan), a peristaltic pump, and a conical tube containing PBS. Perfusion was maintained at 1024 μL min^−1^ by elevating the conical tube to provide additional potential energy, thereby sustaining arterial pressure at 0.8 kPa under continuous monitoring.

A 5-French OCT catheter was introduced through a 3-way stopcock to acquire baseline images of the arterial segment. FM-vesicles (2 mL, 25 µg mL^−1^ in PBS) with gold or DiD were then perfused for 3 min to enable macrophage targeting within plaques via membrane fusion. After resuming perfusion to wash the vessel, OCT images were acquired to visualize plaque structure and vesicle localization, followed by analysis using MicroDicom DICOM Viewer software. The arterial segment was then removed from the perfusion device, and plaque fluorescence was imaged using an IVIS Spectrum system (excitation/emission: 644/665 nm).

### Immunohistochemistry

Vascular tissues were fixed overnight at 4 °C using 10% formalin in PBS and washed three times with PBS. Tissues were embedded in paraffin and sectioned at a thickness of 7 μm. Sections were stained with H&E following standard protocols and imaged using an inverted microscope (DMi8 M, Leica, Wetzlar, Germany). For immunostaining, sections were deparaffinized and rehydrated through a graded series of xylene and ethanol solutions (100%, 95%, 80%, and 70% v v^−1^ in distilled water). Antigen retrieval for MMP9 and CD68 was performed using a high-pH buffer (k800421-2, Agilent Dako, Santa Clara, CA, USA). Endogenous peroxidase activity was blocked by incubating slides in 3% H_2_O_2_ (H1009, Sigma-Aldrich) for 10 min, followed by thorough washing with tris-buffered saline (TBS, ML023-03, Welgene, Gyeongsan-si, Gyeongsangbuk-do, Republic of Korea) and blocking with 5% BSA in PBS.

Slides were incubated overnight at 4 °C with primary antibodies against MMP9 (1:100, NBP2-13173, Novus Biologicals) and CD68 (1:200, NBP2-32831, Novus Biologicals). After three PBS washes, Alexa Fluor 594-conjugated secondary antibodies (1:1000, Jackson ImmunoResearch) were applied for 1 h at RT. Slides were then washed three times with PBS, counterstained with DAPI for nuclear visualization, and imaged by confocal microscopy. Arterial elastin was visualized through its characteristic autofluorescence at 488 nm. Macrophage activation via MMP9 was histologically analyzed through incubation with HRP-labeled secondary antibody (1:5000, anti-mouse polymer, K4001, Agilent Dako) for 20 min at RT, followed by treatment with DAB development solution (K3468, Agilent Dako) for 5 min. After washing with distilled water, nuclei were counterstained using hematoxylin (K8008, Agilent Dako), followed by optical imaging with quantitative analysis using ImageJ.

### Statistical analysis

All analyses were performed using R Studio (R version 4.4.3) and GraphPad Prism (GraphPad Software, San Diego, CA, USA). Categorical results were compared using Fisher’s exact test or the chi-square test, as appropriate. Continuous variables are presented as mean ± standard error of the mean (SEM) for normally distributed data, and as the median with the interquartile range for non-normally distributed data. Two-group comparisons were performed using a two-tailed Student’s t-test for normally distributed data and the non-parametric Mann–Whitney U test for non-normally distributed data. For multiple-group comparisons, one-way analysis of variance (ANOVA) was performed, followed by Tukey’s post-hoc test for pairwise comparisons. Global chi-square scores were calculated for each model, and the incremental prognostic value of plaque slope was evaluated using the likelihood ratio test. Statistical significance was defined as *p* < 0.05. In figures, *p*-values are denoted by asterisks: * for *p* < 0.05, ** for *p* < 0.01, and *** for *p* < 0.001, with dashed lines indicating the specific comparison groups and units specified where applicable.

## Supplementary Information

Supplementary Material 1

## Data Availability

All data supporting the findings of this study, including figures and supplementary information, are available from the corresponding author upon reasonable request. Private clinical information is protected and is not available due to data privacy laws. The python, R, and MATLAB codes are accessible on GitHub via the following link: https://github.com/Sewoom-Baek/OCT and https://mybinder.org/v2/gh/oxylike0830-dot/supplementary-code/main?urlpath=lab/tree/test_1_supplement.ipynb

## Abbreviations

ANOVA: Analysis of variance
BCA: Bicinchoninic acid
BM: Bone marrow
BMI: Body mass index
BSA: Bovine serum albumin
cDNA: Complementary deoxyribonucleic acid
CFD: Computational fluid dynamics
CT: Computed tomography
CTCA: CT coronary angiography
DAB: 3,3′-Diaminobenzidine
DAPI: 4′,6-Diamidino-2-phenylindole
DC-STAMP: Dendritic cell-specific transmembrane protein
DiD: 1,1′-Dioctadecyl-3,3,3′,3′-tetramethylindodicarbocyanine
DiI: 1,1′-Dioctadecyl-3,3,3′,3′-tetramethylindocarbocyanine
DiO: 3,3′-Dioctadecyloxacarbocyanine perchlorate
DLS: Dynamic light scattering
DMEM: Dulbecco’s modified Eagle’s medium
DS: Diameter stenosis
DSPE-PEG: 1,2-Distearoyl-sn-glycero-3-phosphoethanolamine-N-[methoxy(polyethylene glycol)]
EC: Endothelial cell
ESS: Endothelial shear stress
ESSG: Endothelial shear stress gradient
FBS: Fetal bovine serum
FFR: Fractional flow reserve
FITC: Fluorescein isothiocyanate
FM: Fusogenic macrophage
GAPDH: Glyceraldehyde-3-phosphate dehydrogenase
H&E: Hematoxylin and eosin
HEPES: 4-(2-Hydroxyethyl)-1-piperazineethanesulfonic acid
HRP: Horseradish peroxidase
HUVEC: Human umbilical vein endothelial cell
IACUC: Institutional Animal Care and Use Committee
ICAM-1: Intercellular adhesion molecule-1
IL-1β: Interleukin-1 beta
IL-4: Interleukin-4
IL-12: Interleukin-12
IRB: Institutional Review Board
IVIS: In vivo imaging system
LA: Luminal area
LAD: Left anterior descending
LCX: Left circumflex artery
M-CSF: Macrophage colony-stimulating factor
MLD: Minimal lumen diameter
MMP-9: Matrix metalloproteinase-9
MSC: Mesenchymal stem cell
NTA: Nanoparticle tracking analysis
NV: Nanovesicle
OCT: Optical coherence tomography
PBS: Phosphate-buffered saline
PDMS: Polydimethylsiloxane
PE: Phycoerythrin
PFA: Paraformaldehyde
PIEZO-1: Piezo-type mechanosensitive ion channel component 1
qRT-PCR: Quantitative reverse transcription polymerase chain reaction
RBC: Red blood cell
RCA: Right coronary artery
RNA: Ribonucleic acid
RPMI: Roswell Park Memorial Institute
SEM: Standard error of the mean
SMC: Smooth muscle cell
TBS: Tris-buffered saline
TEM: Transmission electron microscopy
TIMI: Thrombolysis in myocardial infarction
TNF-α: Tumor necrosis factor-alpha
VMTK: Vascular Modeling Toolkit
vWF: Von Willebrand factor
