# Supplementary material for: Clinical OCT computation-modeled prognosis of coronary plaque progression with validation by fusogenic macrophage nano-targeting
Source: Nano Converg. 2026 Jul 30;13:38. doi: 10.1186/s40580-026-00567-9 (PMC13424019; doi:10.1186/s40580-026-00567-9)
Supplement: Supplementary file 1 — Supplementary Material 1 [file 40580_2026_567_MOESM1_ESM.docx]

Supplementary Information for

**Clinical OCT Computation-Modeled Prognosis of Coronary Plaque Progression with Validation by Fusogenic Macrophage Nano-targeting**


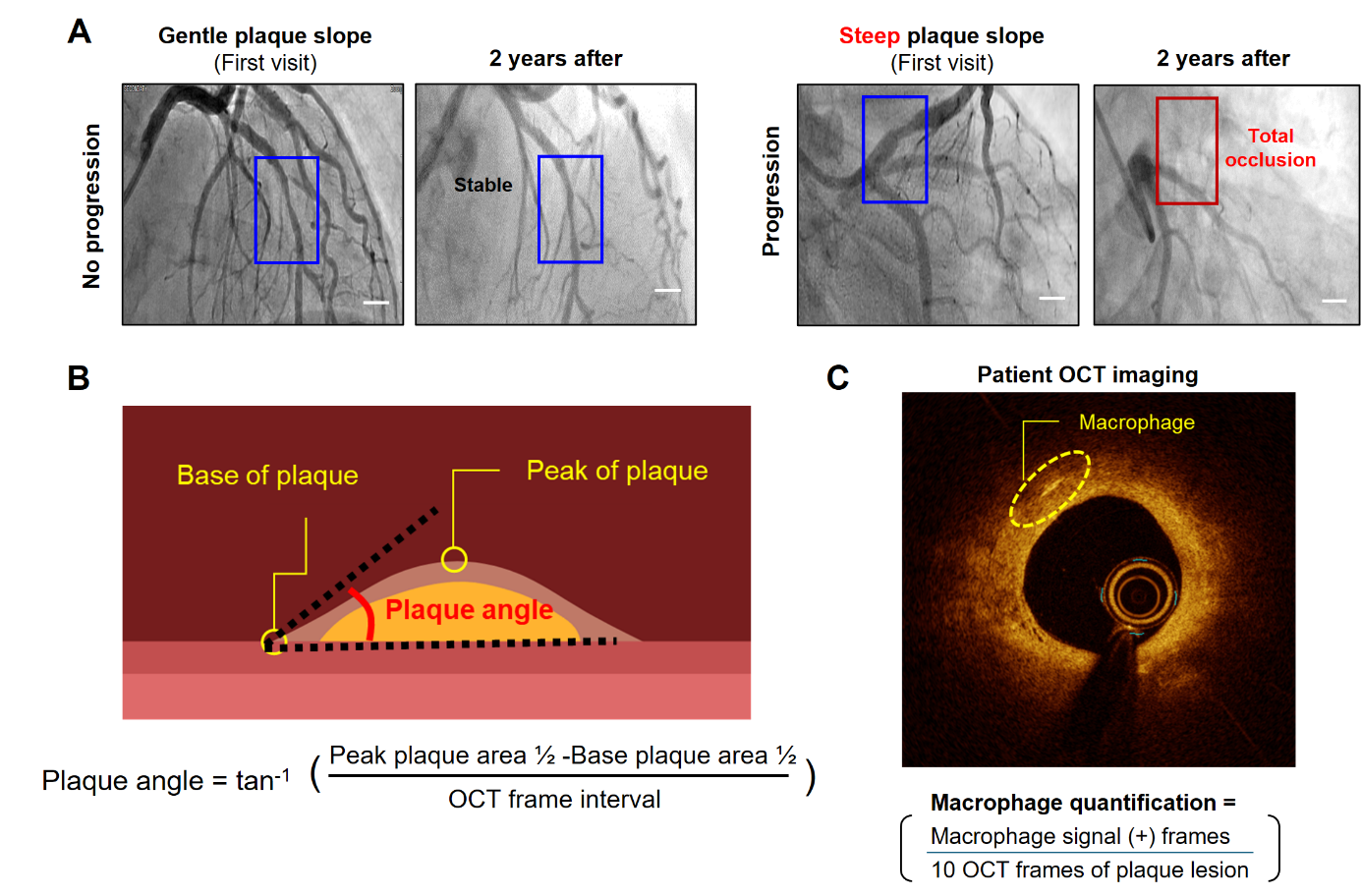


**Fig S1.** **Plaque slope as a clinical prognostic marker of progression and macrophage infiltration in patient coronary arteries.** **(A)** Coronary arteries at the initial visit show a gentle plaque slope (left) that remains patent after 2 years, in contrast to a steep plaque slope (right) resulting in total occlusion (red square) at follow-up (Scale bars = 1 mm). **(B)** Plaque angle is calculated as the arctangent of the area difference between the plaque base and peak, divided by the interval between consecutive OCT imaging frames. **(C)** OCT cross-sectional imaging identifies macrophage-rich regions (yellow circle). The number of macrophage-positive frames within 10 consecutive OCT frames is quantified as a measure of macrophage burden


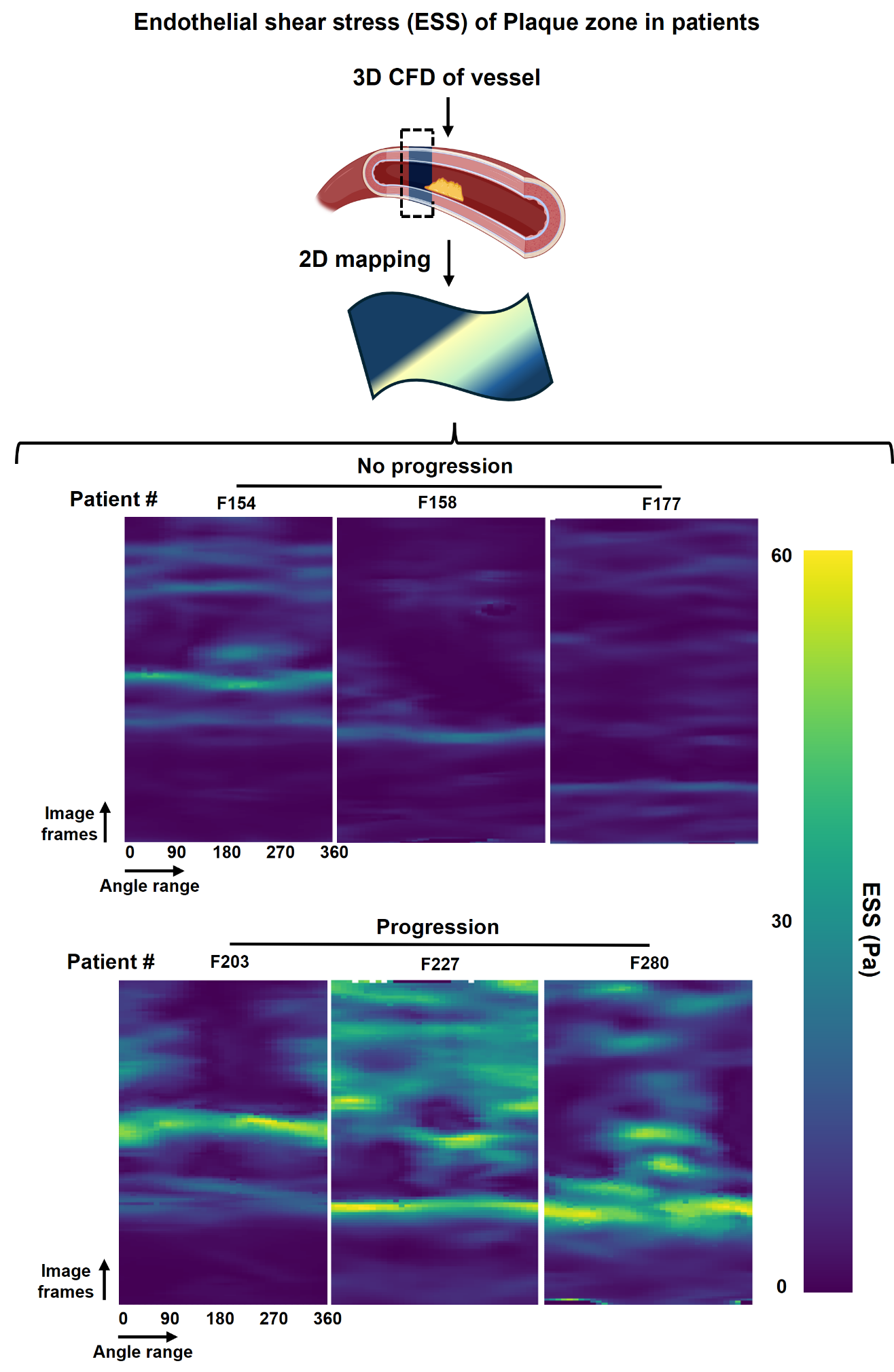


**Fig S2. Patient-specific mapping of ESS distribution in the entry zone of plaque following the flow direction.** 3D computational fluid dynamics (CFD) simulations reconstruct the geometry of each patient’s coronary artery to calculate local ESS within the plaque entry region. The ESS distribution is projected onto the vessel surface and transformed into a 2D heatmap for comparative visualization across patient groups. Non-progressive plaques show relatively uniform ESS with low heatmap intensity, whereas progressive plaques exhibit fluctuating ESS with higher heatmap intensity, reflecting disturbed flow.


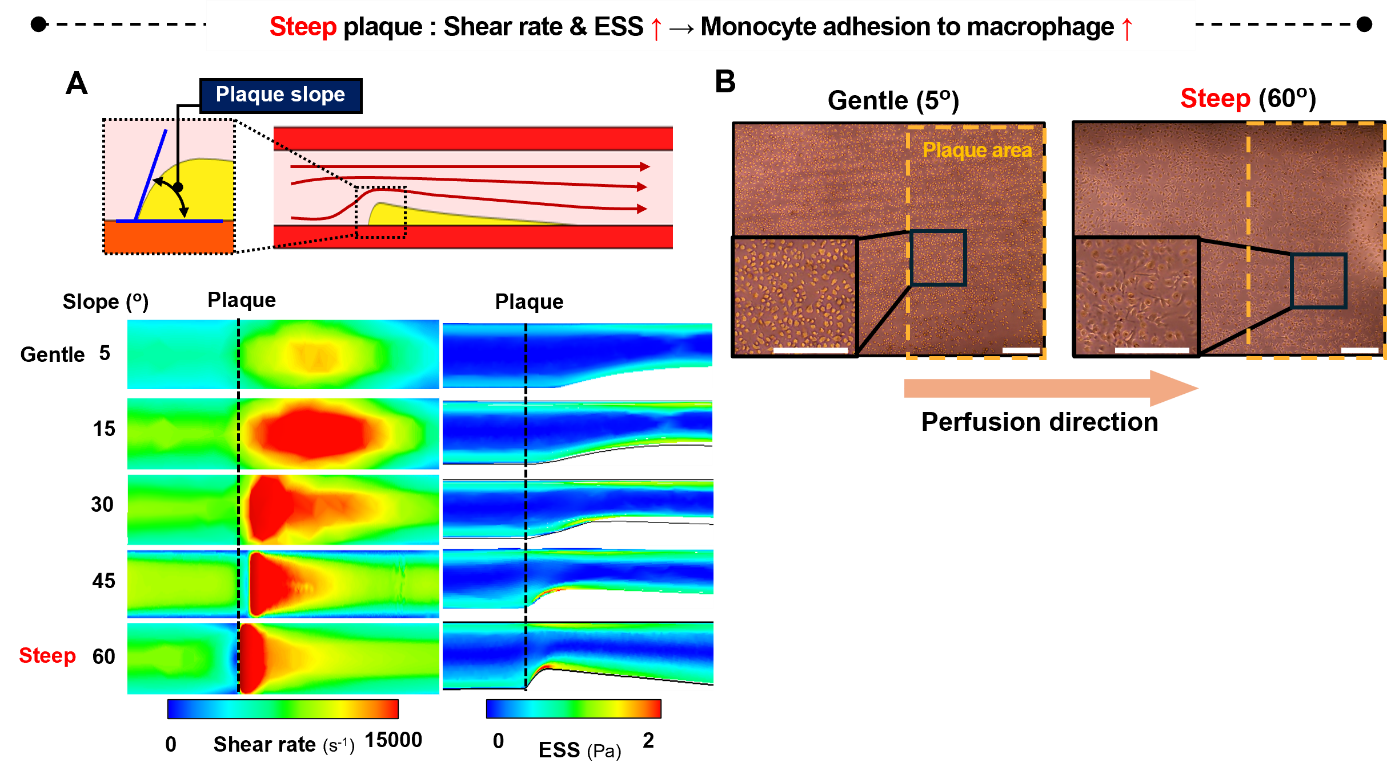


**Fig S3. CFD-Microfluidic modeling to validate plaque slope dependency of shear rate, ESS, and consequent monocyte adhesion.** **(A)** As the plaque slope increases from 5º to 15°, 30°, 45°, or 60° in CFD simulation, the shear rate and ESS increase visibly toward the zone of the plaque region, with the highest levels observed at the steep (60º) slope. **(B)** Monocyte adhesion to macrophages is observed in the plaque area (yellow square) of the plaque-on-a-chip as a microfluidic device model (Scale bars = 100 μm).


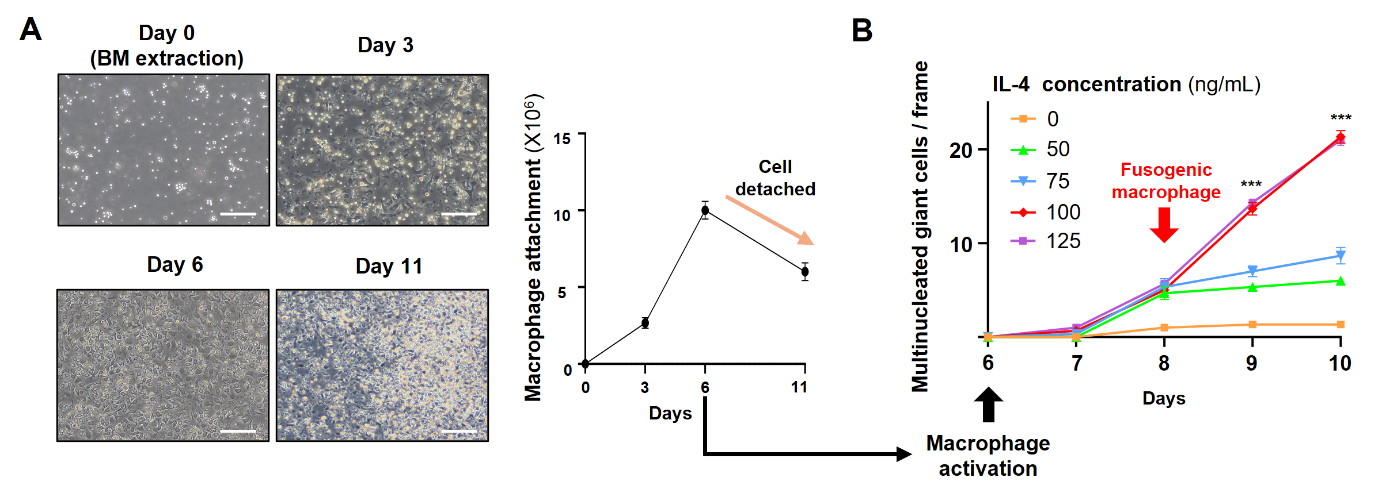


**Fig S4. IL-4 treatment induces fusogenic macrophages by increasing attachment and multinucleation.** **(A)** Monocytes are treated with M-CSF for 6 days to differentiate into macrophages, as indicated by increased attachment and spreading. Subsequent treatment with IL-4 until day 11 induces fusogenic macrophages, which show reduced attachment due to cell fusion and partial cell death (Scale bars = 100 μm). **(B)** IL-4 concentration and timing are evaluated for fusogenic induction. Varying IL-4 doses (0-125 ng mL^-1^) were applied from day 6 to day 11. The optimal time point for fusogenic induction was determined to be day 8, immediately before the exponential increase in multinucleated giant cells. A minimum of 50 ng mL^-1^ administered 2 days effectively induces fusogenic macrophages. Data are presented as mean ± standard deviation (n = 3, independent biological replicates). Statistical significance was determined using a two-sided t-test without adjustment for multiple comparisons. ***p < 0.001 versus the 0, 50, 75 ng mL^-1^ groups.


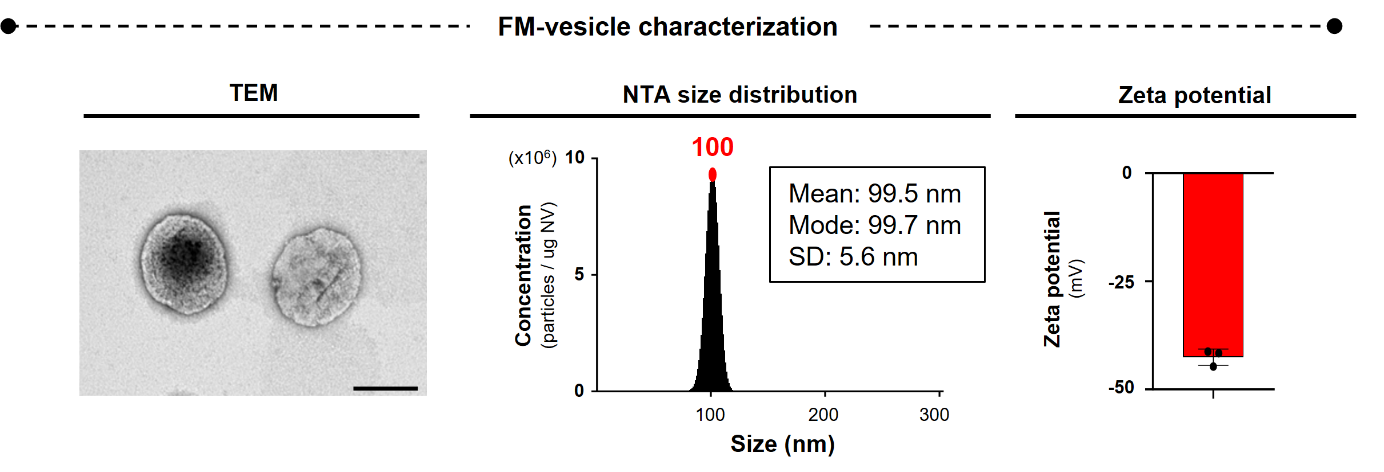


**Fig S5. Size distribution and surface charge of FM-vesicles.** FM-vesicles exhibit typical spherical morphology (left, TEM image, scale bar = 100 nm) with an average diameter of 100-nm (middle, DLS) and a surface charge of -42.51 mV (right, zeta potential).

**
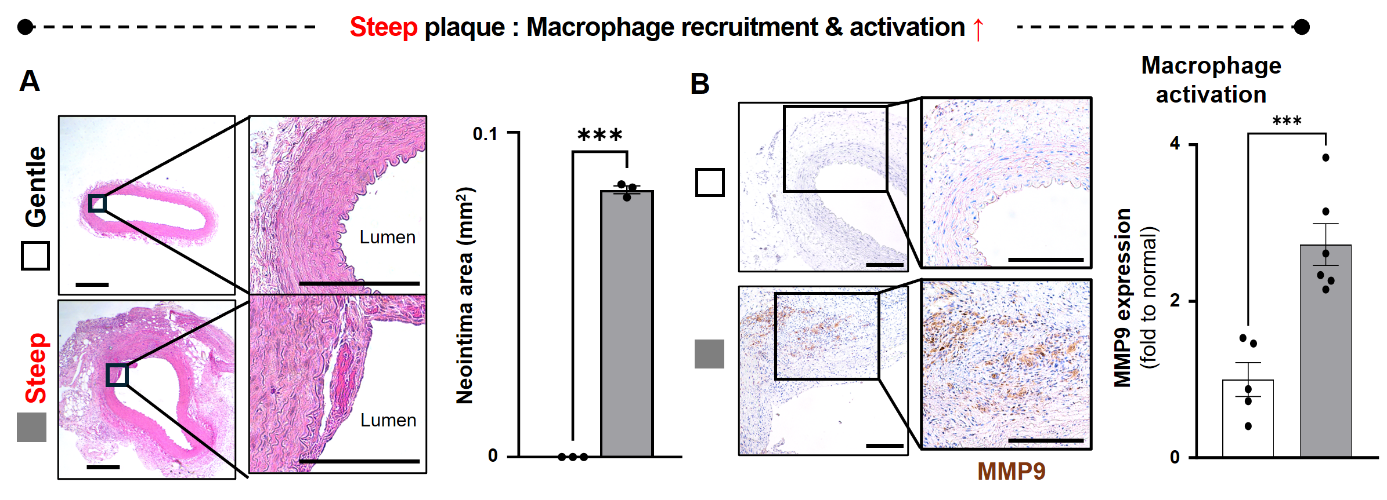
**

**Fig S6. Steep plaque as an inductor of macrophage recruitment with inflammatory activation. (A)** After 28 days following surgical incision, steep plaques exhibit a markedly thickened neointima through vascular remodeling, whereas gentle plaques maintain near normal morphology, as confirmed by quantitative analysis (Scale bars = 400 μm). **(B)** MMP-9 expression is significantly higher in steep plaques compared to gentle plaques, indicating enhanced macrophage recruitment and inflammatory activation (Scale bars = 200 μm). Data are presented as mean ± standard deviation (n: dots represent independent biological replicates in each group). Statistical significance was determined using a two-sided t-test for comparisons between the two groups (***p < 0.001).

**
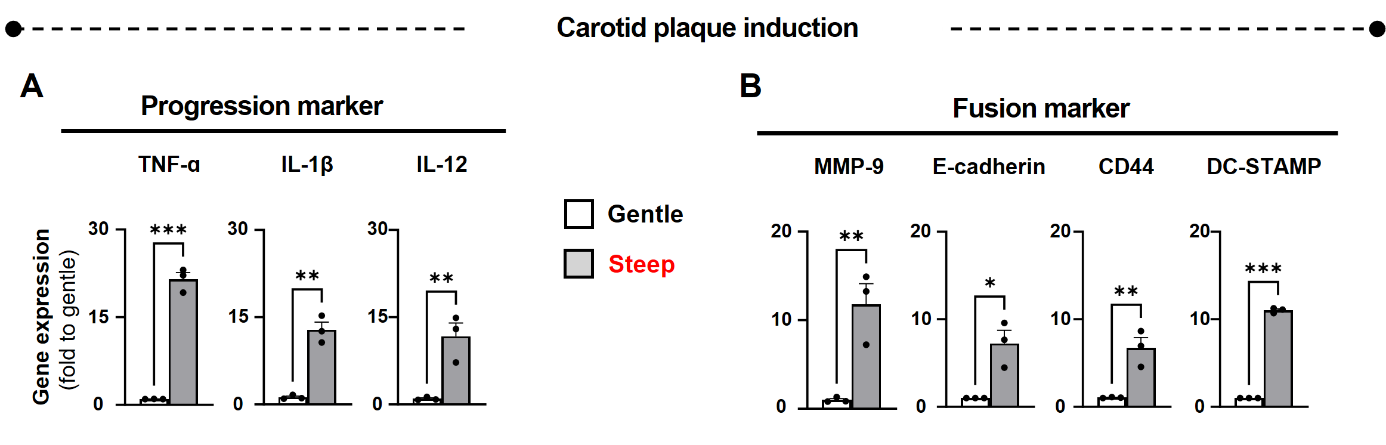
**

**Fig S7.** **Steep plaque as an indicator of stenotic progression with accumulatio of fusogenic macrophages upon incision of rabbit carotid arteries.** **(A)** Compared to the gentle plaques, steep plaques render the artery more vulnerable to stenotic progression as indicated by increased expression of the inflammatory marker genes (TNF-α, IL-1β, IL-12) by PCR analysis. **(B)** Consequently, more macrophages become fusogenic in the steep plaques compared to the gentle plaques, as demonstrated by increased expression of the fusion-related marker genes (MMP9, E-cadherin, CD44, DC-STAMP). Data are presented as mean ± standard deviation (n: dots represent independent biological replicates in each group). Statistical significance was determined using a two-sided unpaired t-test for comparisons between the two groups (***p < 0.05, ****p < 0.01 and *****p < 0.001).

**
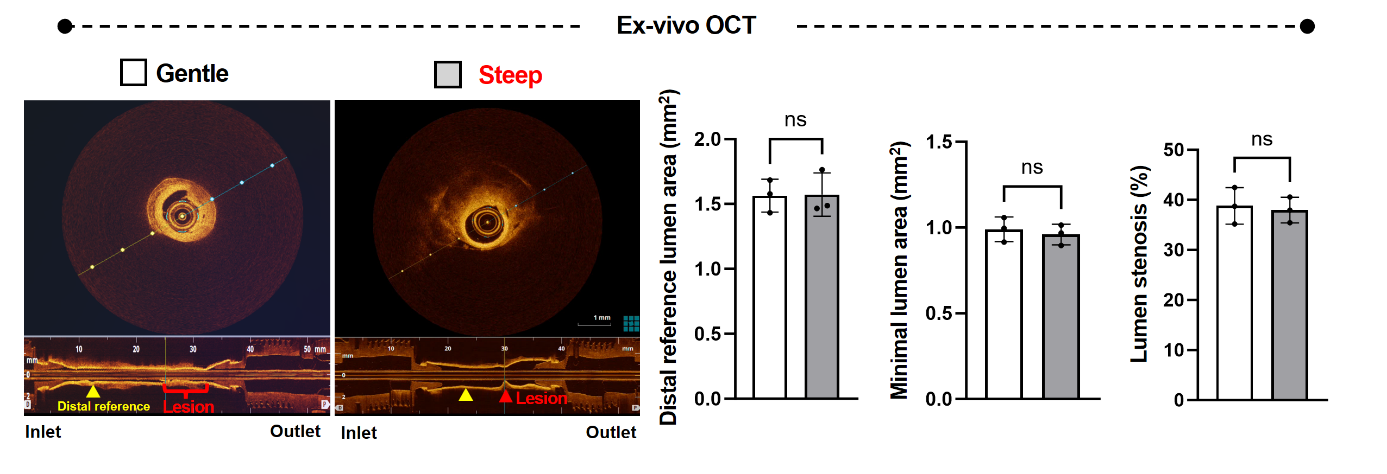
**

**Fig S8. Ex-vivo OCT imaging for luminal geometry analysis of carotid arteries.** OCT images of gentle and steep plaques were acquired ex-vivo to visualize arterial luminal geometry and quantify the distal reference lumen area (yellow), minimal lumen area (red), and the percentage of lumen stenosis. Statistical significance was determined using a two-sided t-test for comparisons between the two groups (not significant).

**
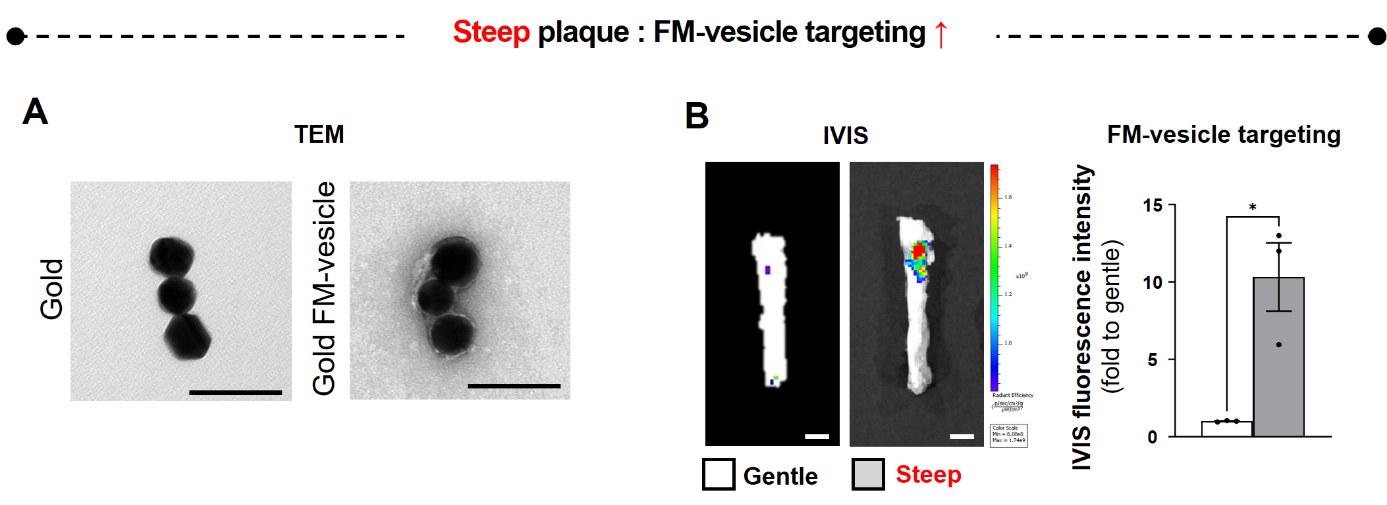
**

**Fig S9. Clinical OCT-based imaging by FM-vesicles with loading gold particles.** **(A)** The Gold nanoparticles remain encapsulated within FM-vesicles after loading, as confirmed by TEM imaging. **(B)** Rabbit carotid arteries were harvested after incision and subjected to IVIS imaging. Steep plaques exhibited significantly higher signal intensity due to targeting by FM-vesicles with DiD, compared to the gentle plaques, as validated by the quantitative analysis (Scale bars = 2 mm). Data are shown as mean ± standard deviation (n=3, independent biological replicates). Significance was determined using a two-sided t-test for comparisons between the two groups (*p < 0.05).
